# Dendritic shaft constrictions shape synaptic integration in neurons

**DOI:** 10.1126/sciadv.aec4911

**Published:** 2026-09-11

**Authors:** Tony Kelly, Michael Döngi, Juan Eduardo Rodriguez-Gatica, Netanel Ofer, Carlos Wert-Carvajal, Niclas Cissewski, Michela Barboni, Philipp Bethge, Christin M. Godale, Sarah Yaser, Michel K. Herde, Jens Tillmann, Sabrina Ingrid Peter, Sebastian Dupraz, Henner Koch, Valentin Stein, Frank Bradke, Steve C. Danzer, Tatjana Tchumatchenko, Martin K. Schwarz, Ulrich Kubitscheck, U. Valentin Nägerl, Heinz Beck

**Affiliations:** ^1^ Institute for Experimental Epileptology and Cognition Research, Medical Faculty, University of Bonn, Bonn, Germany.; ^2^ Institut für Physiologie II, Universität Bonn, Bonn, Germany.; ^3^ Clausius-Institute of Physical and Theoretical Chemistry, University of Bonn, Bonn, Germany.; ^4^ Department of Electrical and Electronics Engineering, Ariel University, Ariel, Israel.; ^5^ Interdisciplinary Institute for Neuroscience, UMR 5297 CNRS, Université de Bordeaux, Bordeaux, France.; ^6^ Brain Research Institute, University of Zurich, Zurich, Switzerland.; ^7^ Department of Anesthesia, Cincinnati Children’s Hospital Medical Center, Cincinnati, OH, USA.; ^8^ Institute of Cellular Neurosciences, Medical Faculty, University of Bonn, Bonn, Germany.; ^9^ Centre for Neuroendocrinology, Department of Physiology, University of Otago, Dunedin, New Zealand.; ^10^ Department of Neurology, Department of Epileptology, RWTH Uniklinik, RWTH Aachen, Aachen, Germany.; ^11^ Axonal Growth and Regeneration Group, German Center for Neurodegenerative Diseases (DZNE), Bonn, Germany.; ^12^ Institute for Neurovascular Cell Biology, Faculty of Medicine, University of Bonn, Bonn, Germany.; ^13^ Department of Anesthesiology, University of Cincinnati, Cincinnati, OH, USA.; ^14^ Institut für Anatomie und Zellbiologie, Universitätsmedizin Göttingen, Göttingen, Germany.; ^15^ Deutsches Zentrum für Neurodegenerative Erkrankungen e.V., Bonn, Germany.

## Abstract

For nearly 150 years, textbooks have described dendritic morphology as optimized for efficient synaptic voltage transfer from spines to the soma, implemented as a tubular design respecting Rall’s 3/2 rule for impedance matching at branch points. Here, we reveal that this view is an oversimplification. Using multiple high-resolution imaging techniques, we demonstrate that dendrites in cortical and hippocampal neurons exhibit nanoscale constrictions, termed dendritic shaft constrictions (DSCs), with diameters ranging from ∼100 to 500 nanometers in mice. We also identified DSCs in human hippocampal and cortical neurons. We provide theoretical and experimental lines of evidence that these constrictions effectively partition the dendrite into distinct electrical compartments, shaping dendritic integration of synaptic potentials.

## INTRODUCTION

Dendrites are the principal recipients of synaptic input in most neurons, integrating thousands of excitatory and inhibitory signals into coherent neuronal output. Their structure is closely linked to function, with dendritic morphology shaping the spatial and temporal dynamics of synaptic integration. Among dendritic specializations, spines—first described by Santiago Ramón y Cajal—represent a canonical nanoscale compartment: a bulbous head connected to the parent dendrite by a thin neck. With diameters on the order of tens to hundreds of nanometers, spine necks create electrochemical compartments essential for synaptic function and plasticity [reviewed in (*1**–**5*)].

In contrast, dendritic shafts are generally conceptualized as smooth, continuous cables. Classical cable theory and its extensions treat dendrites as branching structures that support efficient signal propagation toward the soma, with impedance matching at branch points governed by the 3/2 power law (*6*, *7*). Within this framework, dendrites are modeled as largely uniform conduits whose integrative properties arise from their global geometry, membrane conductances, and synaptic input distribution, rather than from fine-scale structural heterogeneity along the shaft.

This view may partly reflect longstanding limitations in our ability to resolve neuronal morphology at the relevant spatial scale. Whereas dendritic spines are readily identified because of their prominent bulbous heads, nanoscale variations in shaft diameter are considerably more difficult to detect reliably. Constrictions measuring only tens to a few hundred nanometers approach or fall below the diffraction limit of conventional light microscopy and can easily be obscured by optical blur, tissue scattering, labeling inhomogeneities, or reconstruction methods that implicitly smooth neurite contours. Even in electron microscopy (EM) datasets, local caliber variations may have been regarded as incidental irregularities rather than biologically meaningful structures. Consequently, dendritic shaft constrictions (DSCs) may have been overlooked, visually averaged out, or dismissed as fixation, staining, or segmentation artifacts. More broadly, the prevailing assumption that dendrites function as relatively continuous cables may itself have reduced attention toward local shaft irregularities that did not fit existing conceptual frameworks.

At the same time, growing evidence indicates that dendrites support far richer and more localized computations than originally envisioned. Active conductances, regenerative dendritic events, branch-specific plasticity, and clustered synaptic integration have progressively shifted the view of dendrites from passive transmission lines to semiautonomous computational subunits (*6*, *8*, *9*). In this context, dendritic shaft diameter variations could provide an additional, powerful mechanism for compartmentalization within the dendritic arbor and expand the computational repertoire of individual neurons.

Here, we report the identification of localized dendritic shaft diameter variations, which we term DSCs. Using a combination of high-resolution imaging approaches, including expansion microscopy (ExM), stimulated emission depletion (STED) microscopy, image scanning microscopy (ISM), and serial EM, we demonstrate that dendrites in both mouse and human neurons exhibit localized, nanoscale reductions in diameter along their shafts. Data from both computational modeling and two-photon glutamate uncaging suggest that these newly identified structures strongly affect signal integration in the dendrite and may give rise to local signaling compartments along a single dendrite. These findings revise the prevailing view of dendritic shafts as uniform cables and suggest an additional layer of subcellular organization that shapes neuronal computation.

## RESULTS

### DSCs identified with ExM

**Fig. 1.**
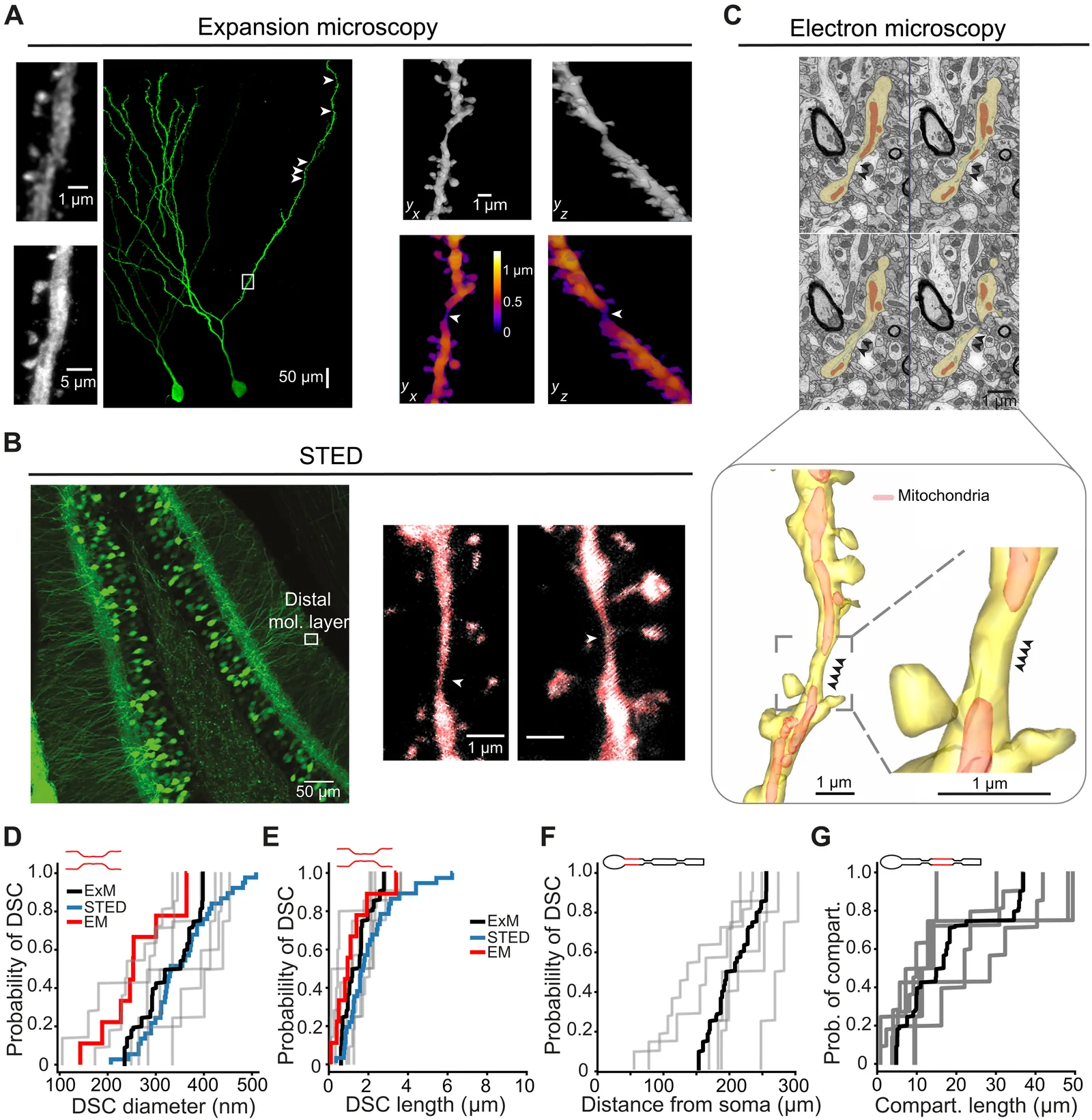
Dendritic nanoscale shaft constrictions in GCs. (**A**) ExM. Left: Light-sheet fluorescence microscopy maximum projection image of GCs sparsely labeled with eGFP. Using ExM, tissue was expanded by 3.8 times to improve resolution. Insets: Far left: Enlargements of same dendritic segment pre- and postexpansion from area indicated with white box in middle panel. Locations of identified DSCs on reconstructed dendrite are indicated by arrows. Right: Two examples of 3D reconstructions as grayscale image (top) or pseudo-colored with the diameter (bottom; see Materials and Methods). DSCs are indicated by arrowheads. Scale bar is corrected for expansion factor. (**B**) STED imaging. Left: Confocal image of dentate gyrus from a Thy1-YFP expressing mouse. Right: STED grayscale images with overlapping binary image (red) of short dendritic segments from the distal molecular layer indicated in left panel. DSCs are indicated by arrowheads. (**C**) EM. Top: Series of four electron micrographs from stack taken from the outer molecular layer (172 μm from GC layer). Segmented dendrite is shown in yellow, DSCs are indicated by black arrowheads, and mitochondria are shown in red. Bottom: 3D reconstruction of the dendrite and enlargement of DSC in inset. (**D** and **E**) Mean cumulative probability curves of DSC diameter (D) and length (E) compiled from the different imaging techniques; ExM (black; individual dendrites in gray), STED (blue), and EM (red). DSC properties were similar across modalities, ExM: DSC diameters of 0.33 ± 0.03 μm (range, 0.11 to 0.46 μm) and length of 1.4 ± 0.2 μm, *n* = 35 DSCs in six dendrites; STED: microscopy DSC diameters of 0.36 ± 0.01 μm (range, 0.21 to 0.51 μm) and lengths of 2.8 ± 0.3 μm, *n* = 24 DSCs; EM reconstructions: DSC diameters of 0.26 ± 0.06 μm and lengths of 1.6 ± 0.7 μm; *n* = 9 DSCs. (**F** and **G**) Cumulative probability curves of DSC distance from soma and dendritic subcompartment size between two DSCs. Data are determined from individual dendrites imaged using ExM.

First, we used ExM in conjunction with a retroviral approach to sparsely labeling hippocampal dentate granule cells (GCs; Fig. 1A). This made it possible to image entire individual dendrites from the soma to their apical tips at high resolution (*n* = 6 dendrites, *n* = 5 cells, and *n* = 4 animals; Fig. 1A and fig. S1, A and B). The average dendritic diameters decreased steadily from proximal (0.80 ± 0.07 μm at 1 to 100 μm from the soma) to distal dendritic segments (0.62 ± 0.08 μm at 100 to 200 μm and 0.54 ± 0.07 μm at >200 μm; fig. S1, B and D). However, we also observed local reductions in dendritic diameter within individual branches where the dendrite is markedly thinner than in the adjacent segments (Fig. 1A, right, and fig. S1, B and C). The local reductions were defined as abrupt drops in dendritic diameter by a factor of 2 or more, which then recovered to at least 75% of the initial dendritic diameter. We termed these structures DSCs. While DSCs were locally defined phenomena, they also diverged from the median branch diameter distribution across the entire dendrite, with an averaged *z*-scored diameter difference of −1.97 ± 0.1 (*n* = 35 DSCs in six dendrites). The average DSC diameter was 0.33 ± 0.03 μm (range, 0.11 to 0.46 μm; *n* = 35 DSCs in six dendrites; Fig. 1D), while their average length was 1.4 ± 0.2 μm (distribution; see Fig. 1E). Repeated imaging did not alter the properties of DSCs (fig. S1, F to H), suggesting that photodamage during imaging does not account for these structures.

Most DSCs were observed distally, with the most proximal DSC being, on average, 207 ± 18 μm (range, 56 to 249 μm; *n* = 6) away from the soma (Fig. 1F and fig. S1B). All GC dendrites we analyzed displayed multiple DSCs when tracking dendrites from the soma to the tip. We counted, on average, five (range, 2 to 10) DSCs per dendrite. Multiple DSCs created inter-DSC compartments, which were, on average, 17 ± 1.5 μm long (distribution; see Fig. 1G). On the basis of basic biophysical principles, these morphological constrictions are expected to increase the axial resistance of the dendrite by more than fivefold, from 5.5 ± 1.9 MΩ to 34.8 ± 6.0 MΩ per μm length of dendrite (*n* = 6 dendrites and 35 DSCs) (*10*), adding a total of 260 ± 114 MΩ in axial resistance (range, 58 to 771 MΩ) to an individual dendrite (*n* = 6 dendrites).

### DSCs identified with super-resolution light microscopy approaches

To confirm the existence of DSCs in live tissue, free of potential fixation artifacts, we used STED microscopy and examined GC dendrites in mice expressing enhanced yellow fluorescent protein (eYFP) in GCs (Fig. 1B, eight slices from three animals). Here, we were not able to reconstruct full GC dendrites. We therefore analyzed multiple dendritic segments from proximal (50 to 100 μm from the GC layer) and distal (>200 μm from the GC layer) dendritic regions (Fig. 1B). DSCs, defined as in the ExM experiments, were readily observed in the STED images (examples shown in Fig. 1B, right, and more examples shown in fig. S1I). As predicted by the distribution of DSCs in ExM experiments, DSCs occurred more frequently in distal (23 of 50) than proximal segments (1 of 15). Their dimensions were comparable to those measured by ExM (see Fig. 1, D and E).

In addition, we used ISM to confirm these live super-resolution results. ISM has lower spatial resolution than STED but offers higher signal-to-noise ratios for a given amount of excitation light. We could readily identify DSCs (fig. S2, A to C) using the Nikon NSPARC system (DSC diameter, 491.2 ± 20.0 nm; DSC length, 2.43 ± 0.18 μm; *n* = 20 DSCs in seven dendrites). Under repeated imaging, the DSCs proved stable, indicating the absence of any photodynamic effects that could explain their presence in the first place (fig. S2D).

### DSCs detected using serial EM approaches

Offering the highest possible resolution for anatomical imaging, we also used EM to detect DSCs in GCs (Fig. 1C). EM images were taken at distal locations (175 ± 24 μm from the soma), and 18 dendritic segments were reconstructed (mean length of segments, 15 ± 0.3 μm; range, 6.3 to 31 μm). Nine DSCs were identified using the same detection criterion used for ExM and STED analyses (in 9 of 18 distal dendritic segments, *n* = 2 mice; Fig. 1C, bottom, and fig. S3, A and B, for more examples). DSC lengths obtained by EM were comparable to values from light microscopy. The distribution of DSC diameters was shifted to lower values, potentially due to the higher spatial resolution of EM (EM, ∼10 nm; ExM, ∼60 nm; STED, ∼40 nm; ISM, ∼100 nm; see Fig. 1, D and E), but the difference between these distributions was not significant (Kruskal-Wallis *H* statistic = 5.9, *P* = 0.053).

In addition, EM images revealed subcellular features of the dendrites, such as tubular structures within DSCs (Fig. 1C and fig. S3B) and mitochondria in adjacent dendritic shafts (fig. S3C). However, absence of mitochondria was not sufficient for a DSC, nor were adjacent dendritic segments simply thicker because of the presence of mitochondria (fig. S3D).

### DSCs are observed in different excitatory neuron types

**Fig. 2.**
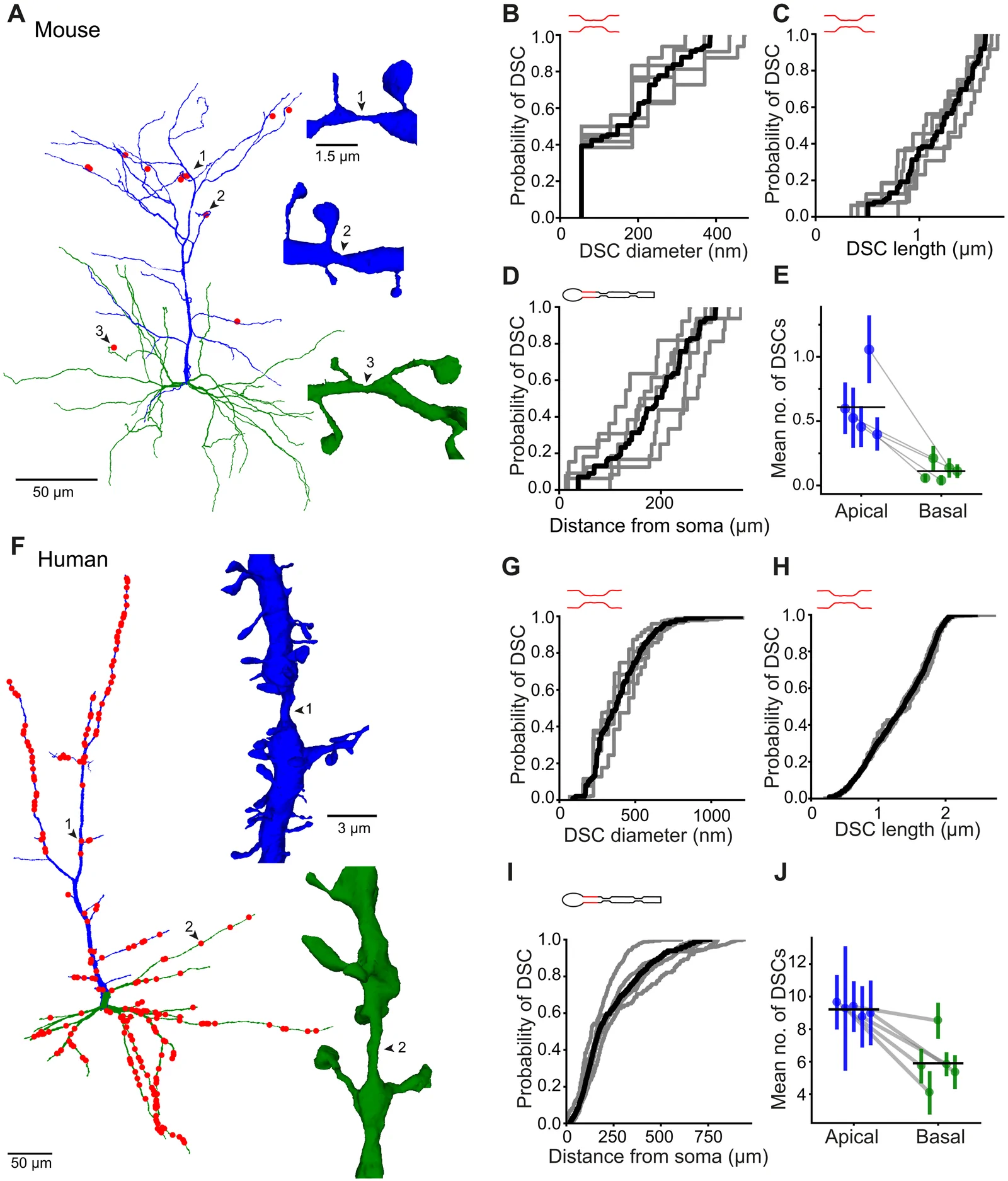
Dendritic nanoscale shaft constrictions in mouse and human cortical dendrites. DSCs are present in mouse cortical pyramidal neurons (A to E). (**A**) Example of an EM reconstruction of an entire layer 2/3 pyramidal neuron, with the locations of the DSCs depicted in the insets indicated with a number. Note the prevalence of DSCs in the apical tuft. Basal dendrites are shown in green, apical dendrites are shown in blue, and DSCs are indicated with red circles. (**B** and **C**) Mean cumulative probability plots of DSC diameter (B) and length (C). (**D**) Cumulative probability plots of DSC distance from soma. Individual neurons are shown in gray; the mean across neurons is shown in black. (**E**) Mean number of DSCs along an entire dendrite from soma to dendritic tip for apical and basal dendrites. Points represent individual neurons (*n* = 5); lines indicate paired measurements. Error bars denote SEM. Statistical significance assessed using Wilcoxon signed-rank test (*P* = 0.0625) and paired *t* test (*P* = 0.013). DSCs also present in human cortical pyramidal neurons [(F) to (J)]. (**F**) EM reconstruction of human cortical layer 2/3 pyramidal neuron with locations of DSCs indicated with red circles. Inserts show example DSCs from apical (blue) and basal (green) dendrites. Mean cumulative probability curves of DSC diameter (**G**) and length (**H**) and distance from soma (**I**). (**J**) Plot of mean number of DSCs along an entire dendrite from soma to dendritic tip for apical and basal dendrites in human cortical pyramidal neurons. Points represent individual neurons (*n* = 5); lines indicate paired measurements. Error bars denote SEM. Statistical significance assessed using Wilcoxon signed-rank test (*P* = 0.0625) and paired *t* test (*P* = 0.0097).

We next investigated whether these structures are specific to dentate GCs. To this end, we examined serial EM from the MICrONS Consortium (*11*) dataset, which contains complete reconstructions of entire cortical pyramidal neurons from layer 2/3 of the mouse visual cortex (Fig. 2A). DSCs were restricted to the fine caliber dendrites with a range of 11 to 16 DSCs in each cell (*n* = 5 pyramidal neurons and *n* = 1 animal; see also fig. S4). DSCs were primarily found in the apical dendritic compartment, with a majority occurring in the distal tufts (DSC locations indicated in a representative neuron in Fig. 2A). In total, 68 DSCs were identified, with 58 in apical and 10 in basal dendrites from five cells (corresponding to ∼85% of DSCs within a cell located in the apical dendrites; Fig. 2A). The average diameter at the DSC was 169 ± 121 nm, ranging between 55 and 476 nm (Fig. 2B), and the average length of the constricted region was 1.2 ± 0.4 μm, ranging between 0.3 and 1.8 μm (Fig. 2C). In keeping with the preferential localization in apical tufts, most DSCs were located distally, at an average distance of 190.3 ± 87.8 μm from the soma, ranging between 13 and 360 μm (Fig. 2D). Apical dendrites exhibited 0.61 ± 0.12 DSCs (means ± SEM) along the path from the distal tip to the soma, compared to 0.11 ± 0.03 in basal dendrites (Fig. 2E). Paired comparison (*n* = 5) showed only a trend with a Wilcoxon test (*P* = 0.0625) but significance with a paired *t* test (*P* = 0.013), indicating limited statistical power due to the small sample size. In addition, synaptic density did not differ between regions immediately adjacent to DSCs (<5 μm) and at least 15 μm away from them, suggesting that the absence or presence of DSCs likely does not affect synapse density (fig. S4, L and M).

We also examined CA1 pyramidal neurons that were fluorescently labeled with enhanced green fluorescent protein (eGFP) and anatomically reconstructed following ExM (fig. S5, A and B). Similar to mouse cortical neurons, no constrictions were observed in the CA1 trunks up to 300 μm from the soma (*n* = 2; *n* = 1 mouse); however, DSCs were also observed in the fine radial oblique dendrites (*n* = 3; see fig. S5, B and C; see DSC diameter and length in fig. S5D).

### DSCs are present in human neurons

A human serial EM dataset (*12*) allowed us to extend our analysis to layer 2/3 pyramidal neurons of human cortex. In these neurons, clear DSCs were observed in both apical and basal dendrites from five cells (Fig. 2F and fig. S6 for further examples). The average diameter at the DSC was somewhat larger than in mouse neurons, 146 nm, ranging between 69 and 841 nm (Fig. 2G), and the average length of the constricted region was 1.27 μm, ranging between 0.12 and 2.91 μm (Fig. 2H). As in mouse cortical neurons, more DSCs were observed in more distal compartments (Fig. 2I), and they were more abundant in apical versus basal dendrites (Fig. 2J). Together, DSCs were consistently observed by EM in three independent preparations from mice and human individuals.

We also found DSCs in two additional human preparations using confocal microscopy (Zeiss Airyscan). Using identical criteria to the analyses in mice, we identified clear DSCs in cortical layer 2/3 pyramidal neurons from epilepsy surgical specimens that had previously been recorded in the slice preparation using patch-clamp approaches, filled with biocytin and stained (fig. S7, A to C; DSC diameter, 406 ± 20.8 nm; DSC length, 3.17 ± 0.33 μm; *n* = 43 DSCs in seven dendrites). Likewise, in human GCs labeled with GFP in organotypic slices (*13*) (fig. S7D), we could also identify DSCs (DSC average diameter, 570 ± 50 nm; average length, 2.3 ± 0.9 μm; *n* = 5 DSCs in three dendrites; fig. S7, E and F).

In summary, using multiple high-resolution imaging approaches based on light and EM, we found a previously unrecognized morphological feature of dendritic shafts in different types of excitatory neurons, in mouse and human brain tissue.

### Computational modeling predicts impact of DSCs on dendritic integration of distal synaptic inputs

**Fig. 3.**
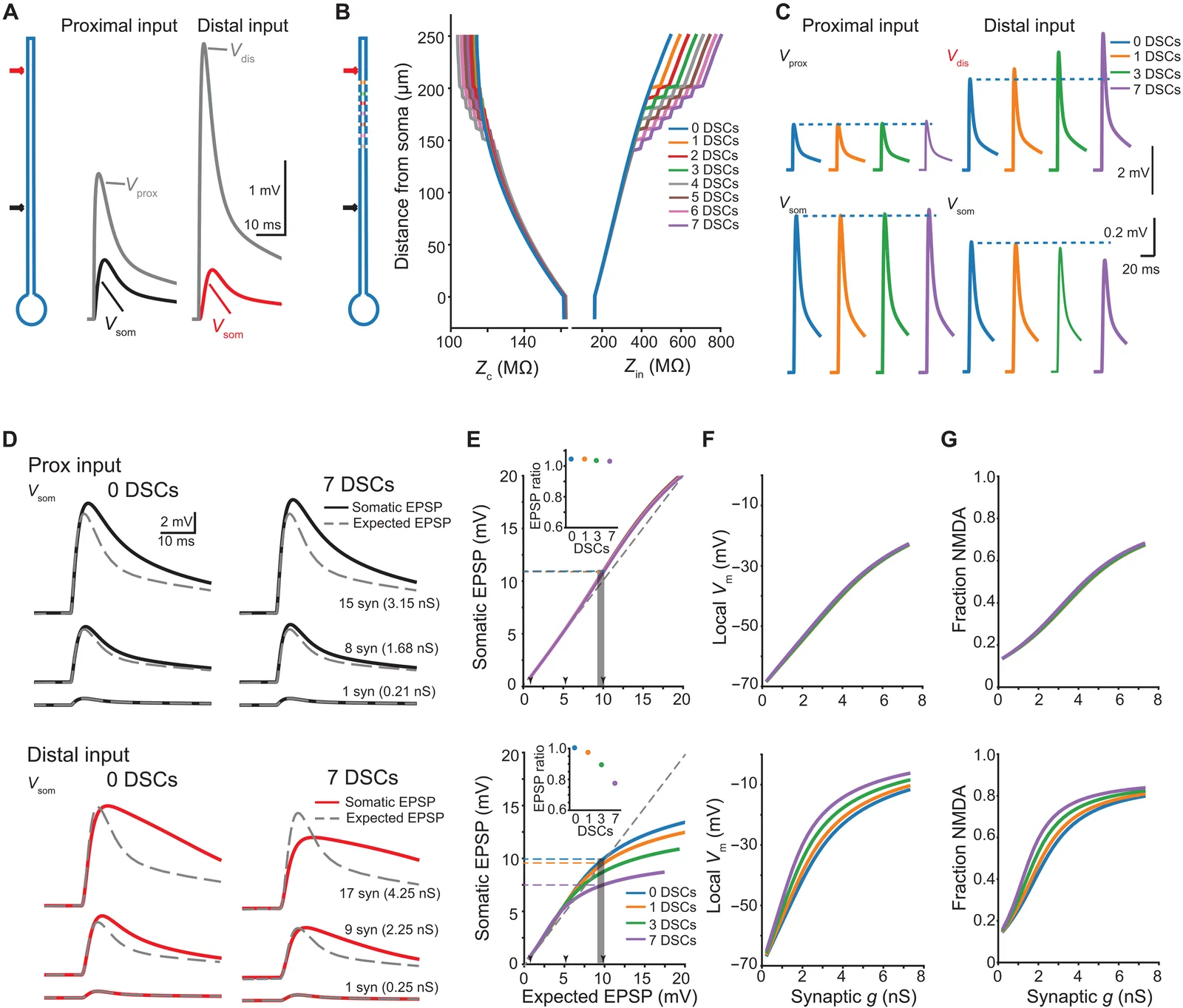
Computational modeling predicts modified dendritic integration of distal inputs. (**A**) Left: Schematic representation of ball and stick model consisting of a soma (diameter, 20 μm) and dendrite (length, 250 μm; diameter, 1 μm), showing position of proximal (black) and distal (red) input. Right: Voltage traces measured at indicated location in response to proximal or distal input, showing attenuation of dendritic EPSP at soma (synaptic conductance *g*_Max_ = 0.26 nS for both 90 and 220 μm from soma). (**B**) Left: Schematic representation of model showing position of one to seven DSCs (length, 1 μm; diameter, 200 nm). Right: Plots showing transfer impedance (*Z*_c_) and input impedance (*Z*_in_). *Z*_in_ increased by 10 to 49% for one and seven DSCs. (**C**) Voltage responses measured at indicated location in response to proximal or distal input with indicated DSCs. (**D**) EPSPs measured at soma in response to increasing input at proximal or distal inputs. Single synaptic conductance was 0.21 and 0.26 nS for proximal and distal sites and set as the conductance to elicit uEPSP amplitudes determined experimentally in fig. S10. Synaptic input increased by increasing synaptic conductance in multiples of the single synaptic conductance. Number of model synapses and total conductance are indicated. Expected EPSP (gray) was determined by scaling the uEPSP by the number of model synaptic inputs. (**E**) plots of somatic against expected EPSP amplitudes for proximal (top) and distal (bottom) input with increasing number of DSCs (lines overlapping with proximal input). Dashed gray lines indicate unity gain. Arrowheads on *x* axis correspond to traces shown in (D). Insets: Mean ratio of somatic EPSP to expected EPSP amplitude at an expected EPSP amplitude of 8 to 10 mV. (**F** and **G**) Plots depicting peak voltage (F) and NMDA receptor recruitment (G) against total synaptic conductance at proximal (top) and distal input (bottom) sites.

To explore the general impact of DSCs on dendritic integration of synaptic inputs, we generated a simplified ball and stick in silico model. Two synaptic input sites were placed, one proximal to where DSCs occur and one near the dendritic tip (90 and 220 μm from soma, respectively; Fig. 3A). Both sites featured mixed AMPA and *N*-methyl-d-aspartate (NMDA) conductances with a 1:1 NMDA/AMPA ratio. Local excitatory postsynaptic potentials (EPSPs) were elicited at each input site (Fig. 3A). We then incorporated one to seven DSCs into the model. Multiple DSCs were incorporated because our ExM experiments showed that multiple DSCs were observed in each individual GC dendrite (average of five DSCs per dendrite). The dimensions of the DSCs in the model were chosen to match their experimentally observed dimensions (200 nm in width, 1 μm in length, and spaced 10 μm apart). This configuration introduced an axial resistance of 58 to 406 MΩ in the model.

The addition of DSCs increased input impedance (*Z*_in_), with *Z*_in_ describing the voltage change occurring for a given current injected at the same location. At the same time, transfer impedance (*Z*_c_) decreased, with *Z*_c_ in this case describing how much somatic voltage change is incurred for a given current injected at a distal input location (Fig. 3B). Consequently, distal synaptic inputs exhibited increased depolarizations at the input site when DSCs were added (increase by 10 to 49%, depending on number of DSCs; Fig. 3C). At the same time, adding DSCs caused an attenuation of somatic EPSPs by 1.4 to 14% with one to seven DSCs, respectively (Fig. 3C, right traces). By contrast, proximal synaptic inputs were hardly affected (Fig. 3C, left traces). Last, we analyzed the scenario where synaptic inputs were placed within dendritic segments bordered by DSCs on both sides (see fig. S8, A and B).

Next, we examined the impact of DSCs on synaptic summation, focusing on somatic voltages up to ∼20 mV (see fig. S8, A and E). For proximal inputs, synaptic summation resulted in largely linear increases in somatic EPSP amplitudes (Fig. 3, D and E, top). The presence of DSCs distal to the proximal synaptic input had little effect on this linear relationship even for large synaptic inputs (Fig. 3, D and E, top and inset).

In contrast, synaptic summation at distal input sites became markedly sublinear as the number of DSCs increased (Fig. 3, D and E, bottom). The EPSP ratio decreased from 1.01 without DSCs to 0.77 with seven DSCs (Fig. 3E, bottom inset). Notably, increasing the unitary synaptic conductance did not alter the EPSP ratio, as the relationship between measured and expected EPSPs remained consistent (fig. S8C). The observed sublinear integration can be attributed to loss of driving force at distal input sites because DSCs, as well as the resulting high input impedance *Z*_in_, boost the local synaptic voltages to the reversal potential of the synaptic conductances (schematic in fig. S8F). DSCs increased both distal dendritic *Z*_in_ and the local membrane depolarization across a range of stimulation strengths to the point where the driving force was substantially reduced (Fig. 3F). The large local voltage changes in the presence of DSCs led to a rapid recruitment of NMDA receptors (Fig. 3G, bottom). These simulations give insights into the potential effects of DSCs on dendritic voltage integration. To assess whether these effects hold in realistic dendritic morphologies, we performed compartmental modeling, using the morphological data obtained from ExM, which allowed the reconstruction of entire dendritic arbors. The reconstructed dendrites (*n* = 3) contained DSCs (between three and five). In these experiments, we then removed DSCs one by one and assessed dendritic signal integration. These experiments confirmed the basic results, where the addition of DSCs leads to large increases in input impedance and sublinear integration for distal synaptic inputs (fig. S9).

### Two-photon glutamate uncaging reveals sublinear summation and enhanced NMDA receptor recruitment at distal GC dendrites

**Fig. 4.**
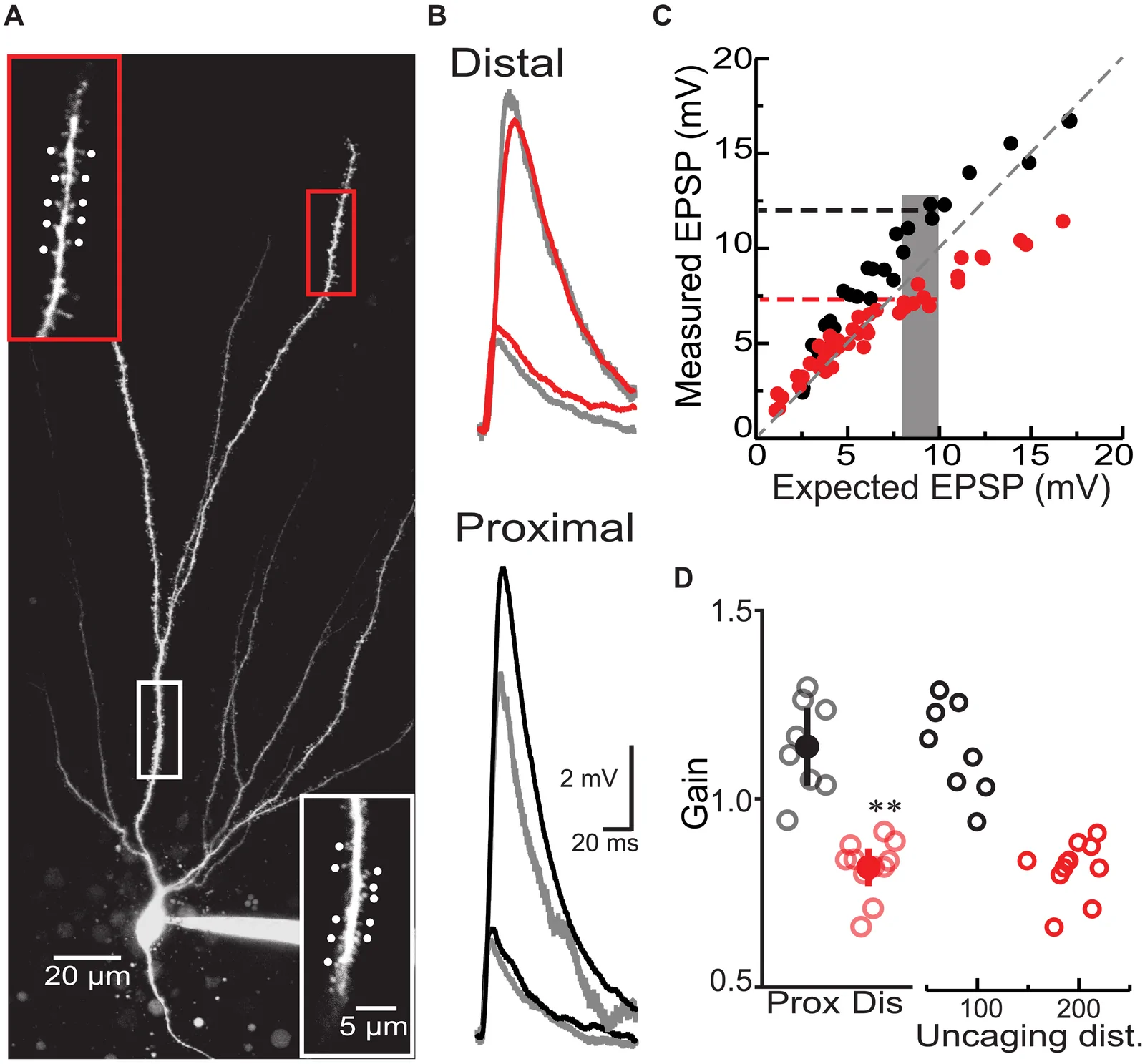
Input site–specific integration of synchronous synaptic input. (**A**) GC filled with Alexa Fluor 594. Two-photon glutamate uncaging was performed at proximal (white box) and distal (red box) input sites. Insets: Higher-magnification images with uncaging points marked by dots. (**B**) Compound EPSPs evoked by near-synchronous glutamate uncaging of 2 and 10 spines at distal (red) and proximal (black) sites. In gray, expected compound EPSPs determined by arithmetically summing individual single spine responses. (**C**) Plots of measured versus expected EPSP amplitudes for proximal (black) and distal (red) inputs for data shown in (B). Dashed gray line indicates unity gain. Gray bar indicates range of expected EPSPs used to compute gain, determined from mean ratio of measured versus expected EPSP amplitudes over 8- to 10-mV range (gray box). Gain of distal input (red dashed line) was lower than gain of proximal inputs (black dashed line) in same cell. (**D**) Summary plot of gain at proximal and distal input sites and with uncaging distance from soma. Statistical significance **P < 0.01 using Mann-Whitney U test between proximal and distal sites.

To experimentally test the predictions of the computational model, we combined two-photon glutamate uncaging either with somatic patch clamp recordings of GCs or calcium imaging in GC dendrites. Two-photon glutamate uncaging was carried out at either proximal or distal sites [78 ± 17 μm versus 195 ± 14 μm from the soma; *n* = 8 and 11, respectively; Fig. 4, A and B; see unitary EPSP (uEPSP) properties in fig. S10]. We found that input summation at proximal sites was linear with a gain above unity, as previously described (Fig. 4, C and D, 1.14 ± 0.10, *n* = 8 dendrites) (*14*), whereas at distal sites, inputs were integrated sublinearly (0.82 ± 0.05, *n* = 11, *P* < 0.01, Mann-Whitney *U* test compared to distal sites), as predicted by the model. Sublinear integration at distal sites was not caused by dendritic expression of K^+^ channels (see fig. S11, A to C).

**Fig. 5.**
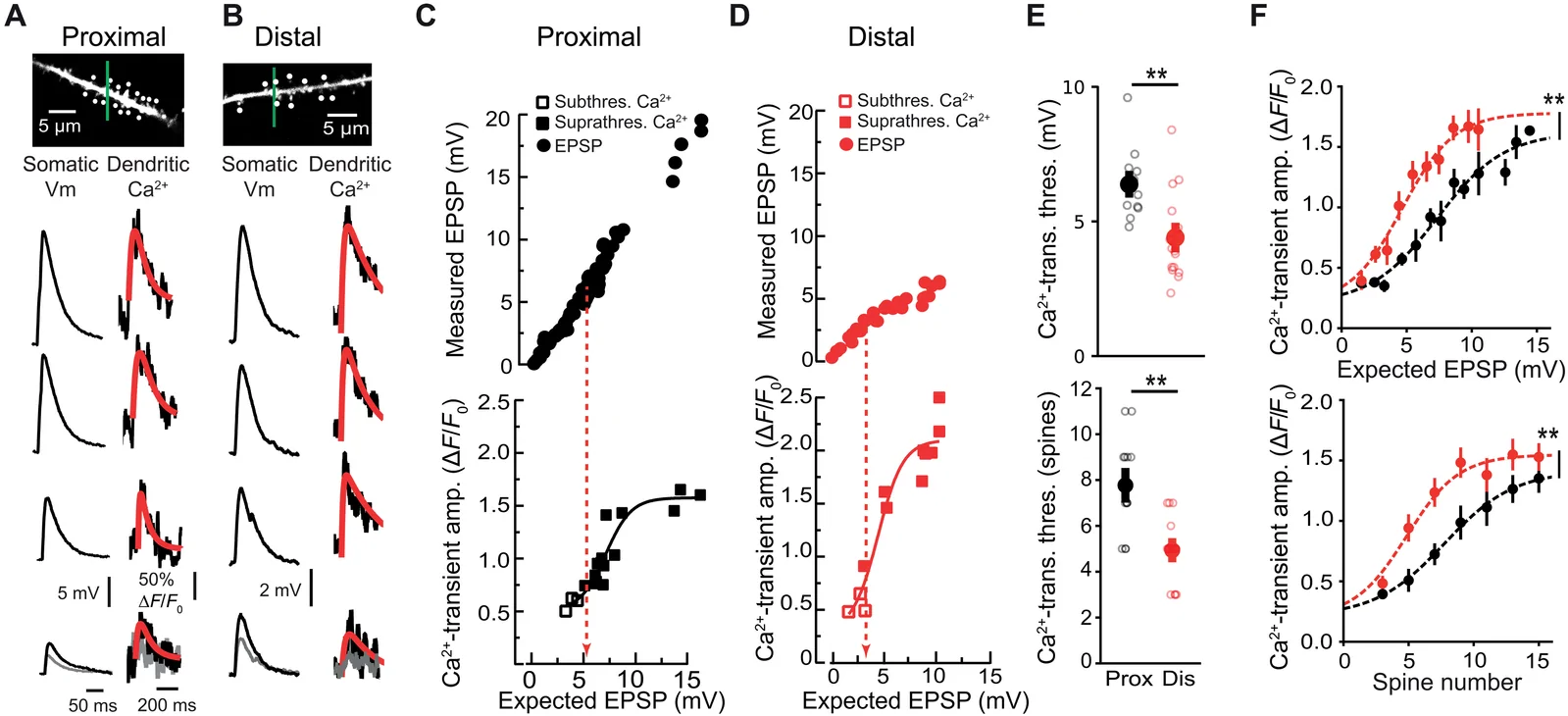
Relationship of local dendritic Ca^2+^ signals and synaptic input at proximal and distal input sites. (**A** and **B**) Two-photon images of GC dendrites with uncaging sites (white spots) and line-scan location (green). Somatic EPSPs and the corresponding local dendritic Ca^2+^ line scans in response to increasing synaptic input at proximal or distal sites. Traces with suprathreshold (black) and subthreshold Ca^2+^ transients (gray). Suprathreshold Ca^2+^ transients were defined by a peak amplitude of >5× the baseline SD and fit with an exponential rise and decay function (red). (**C** and **D**) Plots of measured EPSP amplitudes (circles) and suprathreshold Ca^2+^ transient amplitudes (closed squares) against expected EPSP amplitudes. Subthreshold Ca^2+^ amplitudes are shown in open squares, and threshold is indicated by dashed line. Ca^2+^ responses at proximal and distal input sites fit to a sigmoid function. (**E**) Mean Ca^2+^ transient threshold measured as expected EPSP amplitude (in millivolts) or spine number. Ca^2+^ threshold was significantly lower at distal versus proximal dendrites. Statistical significance determined using unpaired Student’s *t* test distal versus proximal sites, **P < 0.01, for both panels. (**F**) Summary plot showing Ca^2+^-transient amplitude against expected EPSP amplitude or spine number. Mean Ca^2+^-transient amplitudes with SEM binned in 1-mV bins of expected EPSP amplitude or spine number. Ca^2+^-transient amplitude were fit to sigmoid fits. Statistically significant difference determined between proximal and distal site using two-way ANOVA. Main effect proximal (*n* = 4) versus distal (*n* = 9): *F*_(1,4)_ = 10.9, *P* < 0.01; spine number *F*_(1,4)_ = 15.6, *P* < 0.01; interaction: *F*_(1,4)_ = 1.2, *P* = 0.34, and proximal (*n* = 4) versus distal (*n* = 9): *F*_(1,9)_ = 26.8, *P* < 0.01; expected EPSP: *F*_(1,9)_ = 15.9, *P* < 0.01; interaction: *F*_(1,9)_ = 0.4, *P* = 0.93).

We then assessed how steeply dendritic Ca^2+^ signals depend on input strength at proximal and distal sites (Fig. 5, A and B). We found that thresholds to generate Ca^2+^ signals were significantly lower in distal compared to proximal dendrites (Fig. 5, C to E, *n* = 13 and 16 for proximal and distal sites, respectively; Student’s *t* test, both *P* < 0.01), consistent with the modeling results. In addition, Ca^2+^ transients reached saturation with fewer inputs and smaller expected EPSP amplitudes at distal input sites [two-way analysis of variance (ANOVA), *P* < 0.01 for both; Fig. 5, C, D, and F). Blocking NMDA receptors with D-APV significantly increased the thresholds for eliciting Ca^2+^ transients by uncaging (fig. S12, A and B, Student’s *t* test, *P* < 0.01) and shifted the relationship between input and Ca^2+^ transient amplitudes to the right (fig. S12C, two-way ANOVA, *P* < 0.01). These data show that distal dendrites exhibit strong sublinear summation and NMDA receptor recruitment, most likely due to local voltage saturation, as predicted by the computational model.

## DISCUSSION

We report on the discovery of a special morphological feature in dendrites of GCs and other neuronal cell types, both in mouse and human brain tissue, which we termed DSCs. The evidence is based on direct microscopic observations using several different high-resolution imaging techniques, including ExM, STED microscopy, ISM, and EM. We then evaluated the potential functional impact of these constrictions on dendritic integration of synaptic inputs using experimental and modeling approaches.

DSCs exert a strong influence on the passive electrical properties of dendrites and shape dendritic integration. Previous light microscopy studies of GC dendrites reported that step-like decreases in dendritic diameter occur only at branch points (*15*) but lacked the optical resolution to detect DSCs. Even extensive ultrastructural work using EM overlooked DSCs in GCs, likely because these studies tended to focus on the medial molecular layer of the dentate gyrus, where DSCs are less frequent than in distal dendrites (*16*–*18*). Moreover, serial-section EM is labor intensive, and reconstructions are typically restricted to short dendritic segments, reducing the likelihood of detecting DSCs in any neuronal cell type. By contrast, in large high-resolution EM datasets containing entire dendritic arbors, such as those from visual cortex (*11*, *19*), or human middle temporal gyrus (*12*), we could readily identify DSCs.

A key question raised by our findings concerns the functional significance of DSCs for neuronal computation. While our computer simulations and physiological experiments both indicate enhanced local depolarization, sublinear summation, and facilitated NMDA receptor recruitment in distal dendrites, we acknowledge that a direct causal link between DSCs and these physiological effects remains to be established. This limitation arises from the current lack of tools to selectively manipulate the structure or spatial distribution of DSCs. Nevertheless, several lines of evidence support a functional impact of DSCs. First, computational modeling based on both simplified and realistic morphologies indicates that DSCs substantially increase axial resistance and input impedance in distal dendritic segments, creating nonlinear effects on dendritic voltages and Ca^2+^ signals. Second, physiological measurements revealed sublinear summation of synaptic inputs and increased NMDA receptor activation at distal sites that are consistent with these predictions. Mechanistically, an increase in local input impedance is tantamount to boosting EPSPs locally, effectively reducing the driving force for ions entering active synapses, which, in turn, leads to reduced summation of somatic EPSPs. At the same time, increased NMDA receptor activation is bound to lead to increased Ca^2+^ entry and enhanced engagement of Ca^2+^-dependent intracellular signaling cascades. Together, it seems likely that DSCs exert an appreciable influence on the passive electrical properties of dendrites and thus shape dendritic integration.

Beyond their immediate effects on dendritic integration, DSCs may have broader implications for dendritic plasticity. By creating high-impedance subcompartments, DSCs could enable localized nonlinear events, such as NMDA spikes or plateau potentials, thereby supporting branch- or compartment-specific plasticity rules (*20*). Furthermore, DSCs are likely to influence diffusion and active transport, as well as signaling cascades within dendritic shafts, similar to spine necks (*21*, *22*), thereby creating distinct compartments for biochemical signaling. In this framework, DSCs could define functional subunits within dendrites, in which synapses interact cooperatively to drive local plasticity, while remaining largely independent from neighboring compartments. From a theoretical perspective, these mechanisms would implement the ideal of local and parallel signal processing within single dendrites (*20*, *23*). DSCs may also represent a dynamic and regulated feature of dendritic architecture. Future studies combining super-resolution imaging with functional readouts, as well as targeted perturbations of cytoskeletal components, will be critical to determine whether DSCs are developmentally regulated, activity dependent, or altered in disease states. Further intriguing questions that we could not yet address in the present study are, for instance, whether DSCs are regulated during development or under disease conditions or whether they can be modulated by neuronal activity.

An intriguing aspect of our findings is the discovery of abundant DSCs in human cortical neurons, where dendrites are substantially longer and more elaborate than in rodents (*24*). In these extended dendritic arbors, classical cable theory predicts strong attenuation of distal inputs, posing a challenge for effective integration of synaptic signals. The large number of DSCs is puzzling in this respect, as they would be expected to further increase dendritic compartmentalization and synaptic attenuation. One potential explanation to resolve this conundrum is that the high number of DSCs observed in human neurons generates numerous high-impedance compartments that are capable of generating localized supralinear events in response to synaptic inputs. Large local events would be expected to more efficiently propagate toward the soma and contribute to neuronal output. Consistent with this idea, human cortical neurons have been shown to exhibit specialized dendritic nonlinearities that expand their computational repertoire (*25*) and are thought to support a large number of independent nonlinear events, consistent with increased parallel processing capacity of dendritic compartments (*26*). We therefore propose that the high density of DSCs in human neurons contributes to the formation of multiple high-impedance subcompartments along individual dendrites. These compartments may integrate inputs locally and transmit their output to the soma via supralinear events. Although this interpretation remains speculative, it suggests that DSCs represent a nanoscale structural mechanism linking dendritic architecture to species-specific differences in neuronal computation.

A common concern in identifying previously unidentified morphological specializations is the possibility of technical artifacts. We have gone to great lengths to exclude this possibility. We have applied multiple distinct morphological approaches (ExM, STED, ISM, and EM), in both fixed and living tissue, consistently identifying DSCs with similar properties. We also include data from multiple preparations, observing DSCs each time. For instance, we show in three independent EM preparations that DSCs can be readily identified in them. Moreover, their selective dendritic distribution both in mouse GCs and in mouse and human cortical pyramidal neurons supports a genuine biological origin rather than an artifactual one. While we identified DSCs in multiple excitatory cell types in two species (mouse and human), we have not yet analyzed other species, which would shed light on when, during phylogenesis, DSCs first came into being. Likewise, it remains open whether DSCs are also a feature of inhibitory or modulatory neuron types.

Important questions concern the molecular basis of DSCs, which remains unknown. Potential mechanisms include actin-dependent processes linked to periodic cytoskeletal structures found in most dendrites (*27*–*29*) or interactions with surrounding neural elements (*30*–*32*). Identifying these mechanisms will enable targeted manipulation of DSC properties, providing opportunities to probe their roles in neuronal circuits and behavior. In essence, our findings have laid the groundwork for future studies of DSC structure and function.

## MATERIALS AND METHODS

### Animals

All experiments followed institutional guidelines of the Animal Care and Use committee of the University of Bonn and were approved (permission number 81-02.04.2019.A193). All in vitro electrophysiological experiments were performed on adult (8 to 12 weeks) C57BL/6J male wild-type (WT) mice (Charles River, Sulzfeld). For anatomical reconstructions of GCs, C57BL/6J male WT mice were used for EM and ExM and (Thy1-YFP-H mice; the Jackson Laboratory strain 003782) were used for STED microscopy. For reconstructions of CA1 pyramidal neurons, we used WT mice (C57BL/6 mouse line). Mice were kept under a light schedule of 12-hour on/12-hour off, a constant temperature of 22° ± 2°C, and a humidity of 65%. They had ad libitum access to water and standard laboratory food at all times. All efforts were made to minimize animal suffering and to reduce the number of animals used.

### Viral injections

To sparsely label dentate GCs, we injected retroviral constructs derived from a Moloney murine leukemia virus–based vector with GFP expression driven by the chicken β-actin (CAG) promoter [see (*33*)]. WT animals (8 to 12 weeks old) were anesthetized using a mixture of fentanyl (0.05 mg/kg; Janssen-Cilag GmbH, Neuss, Germany), midazolam (5 mg/kg; ratiopharm GmbH, Ulm, Germany), and medetomidin (0.5 mg/kg; Cp-Pharma, Burgdorf, Germany) and placed in a stereotactic apparatus (Neurostar, Tübingen, Germany). Small craniotomies were performed bilaterally at specific coordinates. About 1 μl of retroviral solution was injected (0.25 μl/min) into the dorsal dentate gyri via a Hamilton 1701 RN syringe equipped with a Neuros Adapter and a blunt ended 30-gauge needle (Hamilton, Reno, NE). The following coordinates relative to bregma were used: caudal, 2 mm; lateral, 1.5 mm; and ventral, 2.1 mm. At the end of the procedure, the anesthesia was antagonized by a mixture of naloxon (1.2 mg/kg; ratiopharm GmbH), flumazenil (0.5 mg/kg; Hameln Pharma plus GmbH, Hameln, Germany), and atipamezol (2.5 mg/kg; Orion Pharma GmbH, Hamburg, Germany). Carprofen (5 mg/kg; Pfizer GmbH, Berlin, Germany) was administered as postoperative analgesic, and enrofloxacin (5 mg/kg; Bayer Vital GmbH, Leverkusen, Germany) was administered as antibiotic. Mice were euthanized for immunohistochemistry ∼6 weeks after injection to allow for maturation of GCs.

To sparsely label CA1 pyramidal neurons, we infected the hippocampal CA1 region of WT mice (C57BL/6 mouse line) with an rAAV-expressing eGFP under control of the human synapsin 1 promoter (rAAV-syn-GFP) by stereotaxic virus injection (WPI Benchmark/Kopf; coordinates: rostro caudal, 2.1 mm from bregma; mediolateral, ±1.2  mm from the midline; dorsoventral, 2.1  mm) and stained afterward with an antibody against eGFP. The use of low virus titers (∼10^8^/ml) resulted in a stochastic and relatively sparse labeling of CA1 pyramidal neurons.

### Expansion microscopy

Following viral injection to achieve sparse labeling of CA1 pyramidal neurons or dentate gyrus GCs, tissue was harvested for ExM processing. Animals were deeply anesthetized [xylazine (16 mg/kg of body weight) + ketamine (100 mg/kg of body weight), intraperitoneally; diluted with A. ad inj.; volume of 100 μl/20 g of mouse) and subjected to transcardial perfusion with formaldehyde (4% in PBS). The brains remained in fixative for at least 24 hours before slicing, and slices were prepared at a thickness of 70 to 200 μm using a vibrating microtome (Microm HM 650 V). To ensure uniform labeling and chemical access during the gelation and proteinase digestion phases of the expansion protocol, sections were collected as free-floating slices. The ExM protocol was adopted from (*34*, *35*). Briefly, the labeled hippocampal slices up to 200 μm in thickness were incubated with 2 mM methylacrylic acid–*N*-hydroxysuccinimide linker for 24 hours on a shaker at room temperature (RT). After washing three times in PBS, the slices were incubated overnight (ON) in the monomer solution (8.6% sodium acrylate, 2.5% acrylamide, 0.15% *N*,*N*′-methylene bisacrylamide, and 11.7% NaCl in 1× PBS) on a shaker at 4°C. The gelling solution was prepared by adding 4-hydroxy-TEMPO (0.01%), TEMED (0.2%), and ammonium persulfate (0.2%) to fresh monomer solution. During gelling, the hippocampal slices were placed in a 24-well plate on ice to avoid early polymerization. After applying the gelling solution, samples were put on a shaker at 4°C for 5 min and then transferred to the gelling chamber, followed by 3 hours of incubation at 37°C. After the gel formation, the samples were incubated at 37°C in the digestion buffer [50 mM tris, 1 mM EDTA, 0.5% Triton X-100, 0.8 M guanidine HCl, and proteinase K (16 U/ml) (pH 8.0)], exchanging the buffer every 24 hours if necessary. In general, a 100-μm-thick sample takes about 16 hours to be completely digested. After digestion, the buffer was removed, and the samples were washed three times with PBS. The preservation of the fluorescence of autofluorescent proteins during the expansion procedure enabled us to perform immunolabeling of the GFP-expressing cells after clearing the samples with the digestion.

Immunostaining for GFP was carried out as follows. To prevent unspecific binding of the primary antibody, the samples were incubated with blocking buffer (1× PBS, 5% normal goat serum, 0.3% Triton X-100, and 0.02% sodium azide) on a shaker ON at RT. After blocking, the samples were incubated for 24 hours in blocking buffer with the primary antibody (chicken anti-GFP; 1:500 in blocking buffer; Abcam, ab13970) on a shaker at 4°C. The following day, slices were washed at RT in blocking buffer three times for 30 min and incubated 24 hours in secondary antibody (goat anti-chicken conjugated with Alexa Fluor 488, 1:400 in blocking buffer; A-11039, Thermo Fisher Scientific) on a shaker at 4°C. For nuclear staining, all samples were stained using Hoechst 33342 (H3570, Invitrogen).

Expanded and stained samples were imaged on a custom light-sheet microscope. Briefly, for fluorescence excitation, four fiber-coupled lasers emitting at 405, 488, 561, and 638 nm (Hübner Photonics, Germany) were used. The horizontally scanned light sheet was generated by a galvanometer system with silver-coated mirrors. The adjustment of the beam waist position within the sample chamber was realized by a relay optics mounted on a linear precision stage. The beam waist in the object plane was adjusted to a 1/*e*^2^ diameter of 6.5 ± 0.02 μm for the 405-nm, 7.3 ± 0.02 μm for the 488-nm, 7.0 ± 0.02 μm for the 561-nm, and 8.3 ± 0.02 μm for the 638-nm laser lines. For illumination, we used a Mitutoyo ×10/0.28 numerical aperture (NA) air objective. Our custom-designed sample chamber featured an illumination window formed by a conventional 24 mm–by–24 mm coverslip with a thickness of 0.17 mm. The sample was observed from the top using a Nikon ×25/1.1. NA water immersion objective (CFI75 Apochromat 25XC W) with an additional ×1.5 magnification (Nikon, Germany). The sample was mounted on a coverslip, which could be moved in three spatial directions by motorized microtranslation stages. We used a scientific complementary metal-oxide semiconductor camera (3200 pixels by 3200 pixels, pixel size of 6.5 μm; Kinetix, Teledyne Photometrics, USA) for data acquisition in global shutter mode yielding a pixel size (*x*, *y*) of 0.173 μm and a step size (*z*) of 0.3 in expanded tissue. For analysis, pixel sizes were corrected for the expansion factor of the individual slice (see analysis below). All electronic components were controlled by a custom-written LabView program.

The sample size greatly exceeded the visual field size of 554 μm^2^. Therefore, the data were acquired in a mosaic-like fashion, ensuring a 15% spatial overlap between two neighboring tiles. Complete three-dimensional (3D) representations of the samples were possible after several 3D datasets were stitched together using Fiji (*36*) and the stitching plugin of (*37*). To optimize the stitching process, especially when datasets exceed the available random-access memory of the workstation, the process was performed in two steps. First, substacks of the 3D datasets were created using a Fiji script. Each substack contained about 15% of the information located in the center of the full stack. Second, each substack was stitched to its respective neighboring substack yielding the best overlap in terms of the cross-correlation measure. On the basis of the localization information of each substack after stitching, the full 3D stacks were stitched.

Selected image stacks were spatially deconvolved using Huygens (professional version 22.04, Scientific Volume Imaging, The Netherlands). Deconvolution was performed using theoretical PSFs based on microscopic parameters. The classical maximum likelihood estimation algorithm was used with Acuity: 0.0, and a signal-to-noise ratio value between 8.0 to 20.0 for a maximum number of iterations between 75 to 100 was selected. Stitching and deconvolution were performed on a workstation equipped with two Intel Xeon Gold 6252 CPU (2.1 GHz, 24 cores), 1-terabyte memory, and an Nvidia RTX A4000 GPU (16 GB GDDR6) running under Windows Server 2019.

To assess potential photodamage during light-sheet imaging, we performed a repeated imaging experiment on an expanded mouse brain sample. A 200-μm-thick coronal mouse brain section was prepared from an animal previously injected in the hippocampus with AAV9 hSyn Con/Fon hChR2 (H134R)–eYFP. After perfusion fixation with 4% paraformaldehyde (PFA), the sample was immunostained and expanded as described above. Imaging was performed using a light-sheet fluorescence microscope (UltraMicroscope Blaze, Miltenyi). An overview scan of the hippocampal region was first acquired (volume, ∼9.5 mm by 6.2 mm by 3 mm) to identify a suitable region of interest. A region within the CA1 area containing dendritic structures comparable in size to those analyzed in the study was selected for further analysis. A high-resolution volume (318.5 μm by 592 μm by 712.5 μm) was acquired using a ×25/0.95 NA water immersion objective. This volume was imaged sequentially 20 times, with an interval of ∼20 s between acquisitions, to assess potential photodamage. The analysis was performed on raw data without any processing that could affect morphology or signal intensity (no deconvolution used). Only 3D spatial and temporal registration was applied to correct for drift during acquisition.

### Preparation of acute brain slices

Transgenic animals (Thy1-YFP-H line), 21 to 40 days old, were killed by cervical dislocation, and their brains were quickly removed and placed in ice-cold sucrose-based artificial cerebrospinal fluid (ACSF) containing 210 mM sucrose, 10 mM glucose, 2 mM KCl, 26 mM NaHCO_3_, 1.25 mM NaH_2_PO_4_, 0.1 mM CaCl_2_, and 6 mM MgCl_2_ (pH 7.4; osmolarity, ∼320 mosmol/liter), which was bubbled with carbogen (95% O_2_/5% CO_2_). Sagittal 350-μm-thick hippocampal slices were cut using a vibratome (VT1200, Leica, Mannheim, Germany) and transferred to a heated (32°C) holding chamber with NaCl-based ACSF bubbled with carbogen, which consisted of 124 mM NaCl, 3 mM KCl, 26 mM NaHCO_3_, 1.25 mM NaH_2_PO_4_, 10 mM glucose, 2 mM CaCl_2_, 1 mM MgCl_2_, and 0.6 mM Trolox (pH 7.4; osmolarity, ∼305 mosmol/liter) for 1 hour. Slices were subsequently maintained at RT for a maximum of 4 hours. For the imaging experiments, slices were transferred to a submerged recording chamber, where they were continuously perfused (2.1 ml/min) with ACSF at RT.

### STED microscopy

We imaged the acute hippocampal slices using a home-built STED microscope based on two-photon excitation and STED using pulsed lasers (*38*, *39*). Briefly, a femtosecond mode-locked Ti:sapphire laser (Chameleon, Coherent, Santa Clara, CA) operating at 80 MHz and emitting light at 834 nm was used in combination with an optical parametric oscillator (OPO BASIC Ring fs, APE, Berlin, Germany) to produce STED light pulses at 598 nm that were stretched out to ∼100 ps using a 20-m-long polarization-maintaining fiber. A STED light reflection served to synchronize a second Ti:sapphire laser (Tsunami, Spectra Physics, Darmstadt, Germany) tuned to 910 nm and running at a repetition rate of 80 MHz, which was used for two-photon excitation of the fluorophores. The STED doughnut was created by passing the STED beam through a vortex phase mask (RPC Photonics, Rochester, NY), which imposed a helical 2π-phase delay on the wave front. Wave plates (λ/2 and λ/4) were used to make the STED light circularly polarized before it entered into the objective. The 2P and STED beam were combined using a long-pass dichroic mirror. The two laser beams were moved over the sample in all three dimensions using a *x*-*y* galvo scanner (Janus IV, TILL Photonics) and a *z*-axis nanopositioner (Pifoc, PI, Karlsruhe, Germany). A water immersion objective with a long working distance (1.5 mm) equipped with a correction collar was used (×60 LUMFI, 1.1 NA; Olympus). The correction collar was adjusted to optimize the STED doughnut using gold nanospheres (diameter, 150 nm; BBInternational, Cardiff, UK) and slightly readjusted as a function of imaging depth. The epifluorescence was descanned and imaged onto an avalanche photodiode (SPCM-AQR-13-FC, PerkinElmer, Villebone-sur-Yvette, France). Signal detection and peripheral hardware were controlled by the Imspector scanning software (Abberior Instruments, Göttingen, Germany) via a data acquisition card (PCIe-6259, National Instruments). Regions of interest (20 μm by 20 μm) were imaged with a pixel size of 10 nm and a pixel dwell time of 50 μs. The laser power at the sample was typically around 5 to 20 mW for 2P and 5 to 15 mW for STED.

### Nikon NSPARC system imaging

Dendrites of GFP^+^ and tdTomato^+^ dentate GCs located in the dentate molecular layer were imaged in 40-μm brain sections using a Nikon AXR inverted microscope. Imaging was conducted on archival brain sections from C57BL/6 transgenic mice stored at −80°C from previous studies of GC structure (*40*, *41*). Images were acquired at both low resolution (×100 oil-immersion objective, NA = 1.45; 0.69 μm per pixel; 0.302-μm *z*-step) and high resolution (×100 oil-immersion objective, NA = 1.45; 0.04 μm per pixel; 0.125-μm *z*-step). Laser power and gain for GFP (488 nm) and tdTomato (561 nm) channels were kept constant across all low-resolution images (laser power = 10%; gain = 10). For high-resolution imaging, laser power and gain were increased incrementally by 10 for each successive image (first image: laser power = 10%, gain = 10; second image: laser power = 20%, gain = 20; third image: laser power = 30%, gain = 30). For analysis of potential photodamage, three repeated stacks were acquired, ∼40 min apart on average.

### Electron microscopy

Mice were euthanized and transcardially perfused with 2 to 4% PFA and 2.5% glutaraldehyde in cacodylate buffer. Samples were initially incubated with reduced osmium (1% osmium, 0.8% potassium ferricyanide, and cacodylate buffer) and then washed and incubated in either 320 mM pyrogallol in water or 1% thiocarbohydrazide in water. Afterward, samples went through a second nonreduced osmium staining (1% osmium in water). Then, samples were incubated ON in 1% uranyl acetate in water. Next day, they were incubated in Walton’s lead aspartate. After washing and dehydration, samples were embedded in Epon or Durcupan resin. Serial ultrathin sections (50 nm) were cut on a RMC Powertome and collected on carbon-coated glass slides or formvar carbon-coated copper slot transmission electron microscopy grids. Imaging of the samples was done in a Zeiss Crossbeam 550 with backscattered electron detector or scanning transmission electron microscopy detector, respectively. Consecutive sections were scanned and registered with ATLAS 5 software to gain a z-stack of the same consecutive regions. Each image was 5001 pixels by 5001 pixels with an *x*-*y* pixel size of 10 nm. The serial images were rigidly registered using the Fiji plugin Linear Stack Alignment with SIFT (https://imagej.net/plugins/linear-stack-alignment-with-sift) and then processed using IMOD version 4.11.20 (*42*) to perform segmentation, isosurface rendering, and 3D reconstructions.

### Morphological analysis of cortical pyramidal neurons reconstructed by serial EM

Mouse neurons were downloaded from the MICrONS dataset (*11*) and included these neuron IDs: 864691135693733567 (Fig. 2A), 864691136194996812, 864691135374673737, 864691135738411121, and 864691134884807418 (version 943). Human neurons were obtained from the H01 dataset (*12*), with neuron IDs: 3896803064, 4083797526, 4377103473, 5629416191, and 41207002060. Neuronal skeletons were generated using Ultraliser (*43*) with a segment resolution of 75 nm. DSCs were automatically detected on the basis of the following criteria: a local reduction in dendritic shaft diameter of at least twofold, followed by a recovery to at least 75% of the original diameter within a distance of no more than 3 μm. In addition, the absolute reduction in diameter was required to exceed 500 nm. In the mouse tissue, all detected DSCs were manually validated using the 3D dendritic reconstruction. Because of the number of automatically detected DSCs in the human cortical tissue, not all DSCs were manually validated. The analysis code is available on GitHub.

### Analysis of biocytin-labeled human cortical neurons

Cortical neurons were biocytin-filled during whole-cell patch-clamp recordings described previously (*44*). Briefly, slices were transferred to the recording chamber and continuously perfused with carbogenated (95% O_2_ and 5% CO_2_) recording artificial cerebrospinal fluid (125 mM NaCl, 25 mM NaHCO_3_, 2.5 mM KCl, 1.25 mM NaH_2_PO_4_, 1 mM MgCl_2_·6H_2_O, 2 mM CaCl_2_, and 25 mM glucose) at 30° to 33°C. The intracellular patch solution contained 140 mM K-gluconate, 1 mM CaCl_2_, 10 mM EGTA, 2 mM MgCl_2_, 4 mM Na_2_ATP, 10 mM Hepes (pH 7.2) (KOH/HCl), an osmolarity of 300 mosmol/liter, and biocytin (5 mg/ml; B4261, Sigma-Aldrich). Thin-walled borosilicate glass capillaries (3 to 5 MΩ) were used, with recordings maintained for at least 20 min to ensure complete cell filling. Slices were fixed ON in 4% PFA (neutral-buffered) at 4°C. Immunohistochemical processing followed the same protocol as described before (*44*), slices were washed three times for 10 min each in PBS + 0.3% Triton X-100 + 0.3% Tween 20 (PBS-T-T), incubated ON at 4°C in PBS-T-T with streptavidin-Cy3 (1:100, 1 mg/ml; Sigma-Aldrich), washed again three times for 10 min in PBS, and mounted with Fluoromount-G (Invitrogen) or VECTASHIELD (Vector Laboratories, H-1400). Confocal imaging was performed on a Zeiss LSM980 Airyscan as described previously (*44*). Neurons were manually located and focused with a ×10 objective, and imaging regions were defined for each neuron. Fluorescence was excited at 488 nm (Alexa Fluor 488) and 548 nm (Cy3) using a tunable white laser, with emission detected at 520 and 555/615 nm, respectively. After capturing a dendritic overview image, segments of at least 40 μm were selected for the dendritic analysis. Z-stacks were acquired with a ×40 objective at 0.22-μm step size to capture the full dendritic extent. Segments were chosen to span as few focal planes as possible to minimize imaging and laser exposure time. Laser power and gain were adjusted to ensure clear visibility of the fine structure of the dendrites, accepting slight oversaturation of the dendrite shaft where necessary.

### Confocal microscopy of human organotypic hippocampal brain slice cultures

The preparation and cultivation of human organotypic hippocampal brain slice cultures were carried out according to the protocol described previously (*13*). At 1 day in vitro (DIV), the slices were transduced with 1 μl of rAAV-hSyn-eGFP (#50465-AAVrg, Addgene). At DIV 15, the slices were fixed in 4% PFA and mounted in Fluoromount-G (SouthernBiotech). Confocal imaging was performed using an inverted Zeiss LSM 980 confocal laser scanning microscope with Airyscan 2, along with the Zeiss ZEN blue 3.6 software with the ZEN Module Airyscan Joint Deconvolution. Overview images of the dentate gyrus were taken using the Plan-Apochromat M27 ×10 (0.45 NA) objective with a laser wavelength of 488 nm. For detailed imaging, regions with a high fluorescence level in sparsely populated areas of the medial-distal part of the apical dendrite were selected. Z-stacks were captured with a laser wavelength of 488 nm using the Plan-Apochromat ×40 (1.3 NA) oil-immersion objective with a 4× zoom and a step size of 0.18 μm. The images underwent further processing using the Airyscan processing and joint deconvolution with 10 iterations, as provided by Zeiss ZEN blue 3.6 software.

### Analysis of DSC morphology

Individual dendrites were binarized and dendritic diameters determined in Fiji using the Local Thickness plugin. Local Thickness was extracted at the center of the dendrite using a skeleton created from the binarized image using the Fiji plugin Skeletonize (2D/3D) (*45*). Fluorescent images of dendrites were filtered to reduce noise using the 3d median filter plugin (*46*) with a kernel radii of 1, 1, and 0.2 in pixels. The images were binarized using a threshold automatically set using the MaxEntropy method in Fiji and adjusted manually if necessary. Open holes were closed using two to three iterations of Dilate, followed by Fill Holes and the Erode functions. Following binarization, all binary images were superimposed on original grayscale images, overlap was manually compared, and deviations were corrected. Binary images were scaled such that the *x*, *y*, and *z* scales were identical using the scale plugin in Fiji and missing places were interpolated using the bicubic average process. Using the Local Thickness plugin, binarized images were filled with the largest nonoverlapping spheres (fig. S1B). The binarized image was also smoothed with 3D Gaussian filters and skeletonized to provide a path through the middle of the dendrite that was used to extract the diameter of the largest sphere at each location along the dendrite using MATLAB. Data were then analyzed in Python. The extracted diameters were smooth using a boxcar filter over 10 pixels. DSCs were identified as drops in diameter that were a factor of 2. Edges of the constrictions were where the sharp changes in first derivative of the diameter returned to values below 20 nm per pixel. The diameter had to recover by at least 75% of the original value. In the case of ExM, diameters were corrected for expansion. The expansion factor was experimentally determined for each tissue section by comparing somatic and dendritic images obtained before and following expansion (see fig. S1A). Preexpansion images were enlarged by 3.95 +/− 0.16 times (average +/− SD) (range, 3.8 to 4.1) to optimally overlap with postexpansion images. The axial resistance due to morphological changes were calculated as described by Tønnesen *et al.* (*10*) using the following formula

$$R_{\text{morph}}=\frac{\rho L}{A}$$

The sum total change in axial resistance for the entire dendrite was calculated by adding resistance of all DSCs along the dendrite.

### Electrophysiology-slice preparation and patch-clamp recording

Mice were deeply anesthetized with isoflurane and then decapitated. Brains were rapidly removed and placed in ice-cold (<2°C) sucrose-based ACSF containing 60 mM NaCl, 100 mM sucrose, 2.5 mM KCl, 1.25 mM NaH_2_PO_4_, 26 mM NaHCO_3_, 1 mM CaCl_2_, 5 mM MgCl_2_, and 20 mM glucose. Horizontal slices of 300 μm were cut with a vibratome (Leica VT1200S, Wetzlar, Germany) and incubated in sucrose-based ACSF at 35°C for 30 min. Subsequently, slices were transferred to a submerged holding chamber containing normal ACSF containing 125 mM NaCl, 3.5 mM KCl, 1.25 mM NaH_2_PO_4_, 26 mM NaHCO_3_, 2 mM CaCl_2_, 2 mM MgCl_2_, and 15 mM glucose at RT. All extracellular solutions were equilibrated with 95% O_2_ and 5% CO_2_.

Cells within the suprapyramidal layer were visualized with infrared oblique illumination optics and a water immersion objective (×60, 0.9 NA; Olympus). All cells were clearly in the GC layer, and we targeted cells in the first third of the GC layer that displayed a more oblong shape to record from cells with apical and basal processes in plane with the slice surface to ensure that dendrites were not cut. Somatic whole-cell current-clamp recordings were performed with a BVC-700 amplifier (Dagan Corporation, Minneapolis MN, USA). Data were filtered at 10 kHz and sampled at 50 kHz with a Digidata 1440 interface controlled by pClamp software (Molecular Devices, Union City, CA). Patch pipettes were pulled from brosilicate glass (outer diameter, 1.5 mm; inner diameter, 0.86 mm; Science Products, Hofheim, Germany) with a Flaming/Brown P-97 Puller (Sutter Instruments, Novato, USA) to resistances of 2 to 5 MΩ in bath and series resistances ranging from 8 to 30 MΩ. Only cells with an input resistance of <400 MΩ were selected to exclude recordings from newly generated GCs (*47*). The standard internal solution contained 140 mM K-gluconate, 7 mM KCl, 5 mM Hepes, 0.5 mM MgCl_2_, 5 mM phosphocreatine, and 0.16 mM EGTA. Internal solutions were titrated to pH 7.3 with KOH, had an osmolality of 295 mosmol, and contained 50 to 100 μM Alexa Fluor 594 (Invitrogen, Eugene, OR, USA). In experiments to record synaptic-induced intracellular Ca^2+^ changes in the dendrite, Cal-520 (200 μM; Invitrogen) replaced EGTA, and recordings were begun following 20 min to allow equilibration of the dye in the cell. Voltages were not corrected for the calculated liquid junction potential of +14.5 mV. Membrane potential was adjusted to −70 mV for all recordings. Active and passive properties were determined from hyperpolarizing and depolarizing current steps (800 ms) of increasing amplitudes injected via the somatic patch pipette. Passive membrane properties, action potential properties, and firing patterns were assessed throughout the entire course of the experiment. Cells with unstable input resistances or lacking overshooting action potentials were discarded, as well as recordings with holding currents of >−200 pA for 70 mV and access resistances of >30 MΩ.

### Two-photon uncaging

Two-photon glutamate uncaging at apical dendrites of dentate GCs was performed using a dual galvanometer-based scanning system (Prairie Technologies, Middleton, WI, USA) to photorelease glutamate at multiple dendritic spines. MNI-caged l-glutamate (15 mM; Tocris Cookson) was dissolved in Hepes-buffered solution [140 mM NaCl, 3 mM KCl, 2 mM MgCl_2_, 2 mM CaCl_2_ 20 mM d-glucose, and 10 mM Hepes (pH 7.4) adjusted with NaOH (305 mosmol/kg)] and was applied using positive pressure via glass pipettes (<1 MΩ) placed in closed proximity to the selected apical dendrite. Distance measurements were performed on the maximum projection of image stacks collected at the end of recordings using Fiji (ImageJ, NIH). The distance between the soma and the input site was measured as the length of line from the center of the soma along the dendrite to the approximate midpoint of the input site. All uncaging locations included in this study were located >25 and <200 μm from the soma. We used two ultrafast laser beams of Ti:sapphire pulsed lasers (Chameleon Ultra, Coherent). One pulsed laser tuned to 820 nm to excite the Alexa Fluor 594 and select 10 to 15 dendritic spines in close vicinity (∼10 μm in length); another tuned to 730 nm to photorelease MNI-glutamate at the preselected spines. The intensity of each laser beam was independently controlled with electro-optical modulators (Conoptics Model 302RM, Danbury, CT, USA). MNI-glutamate was uncaged at increasing number of spines (*2*–*15*) with a 1-ms exposure times, and the laser was rapidly moved from spine to spine with a transit time of ∼0.1 ms. In any group of spines, uncaging was always performed from the most distal toward the most proximal spine. The laser power at the slice surface was kept below 22 mW to avoid photodamage. The midpoint of the stimulated dendritic region was assessed using maximum projection images from two-photon stacks, and the 2D distance was determined.

The glutamate was uncaged onto a sequence of single spines to elicit uncaging evoked uEPSPs. To quantify deviations from linearity in dendritic integration, the arithmetic summation calculated from each individual single-spine uEPSP was compared to the actually measured compound EPSP during glutamate uncaging onto the same sequence of spines.

### Ca^2+^ imaging

The calcium dye Cal-520 (200 μM) was loaded into the cell via the patch pipette and the cell filled for at least 20 min after break-in before recording. Cal-520 was used for all experiments, as we found that Cal-520 had higher signal to noise compared with or OGB-1 (200 μM; see fig. S13). Two-photon imaging of Cal-520 and Alexa Fluor 594 (50 μM) was performed using a pulsed laser tuned to 820 nm, the fluorescent emissions were collected via a water-immersion objective (×60, 0.9 NA; Olympus), and signals were separated by a dichroic mirror (DXC 575). Red Alexa Fluor 594 emission collected at 605/45 and green Ca^2+^ dye emission collected at 525/70 were detected using separate photomultiplier tubes (Hamamatsu). With these filters, red Alexa Fluor 594 emission was not detected above noise in the green channel. Simultaneous whole-cell somatic recording and local dendritic calcium imaging (line scans at 425 to 750 Hz) were conducted. For action potential induced Ca^2+^ transients, APs were triggered by one to five short current injections (2 ms) in a train (at 100 Hz) through the patch pipette. Each trial was repeated at least three times, and the mean value is presented. For uncaging evoked EPSP-induced Ca^2+^ transients, the line scan was centrally placed between spine number 3 and 5. To reduce photodamage during simultaneous two-photon Ca^2+^ imaging and uncaging, line scans were performed in response to uncaging on 3, 5, 7, 9, 10, 12, 14, and 15 spines. In addition, we used a passive pulse splitter to convert each uncaging laser pulse into eight subpulses to further reduce photodamage (*48*). For iontophoresis-induced Ca^2+^ transients, the line scan was placed across the proximal dendrite near the iontophoretic pipette. Intradendritic Ca^2+^ transients are reported as changes in Cal-520 fluorescence (Δ*F*/*F*_0_), normalized to baseline fluorescence (*F*_0_) averaged over the 190-ms period before stimulation. Calcium transients were distinguished from noise as a fluorescence peak at least 4× above the SD of the baseline period and were fit by a double exponential with a rise tau within the range of 1 to 50 ms and a decay tau within the range of 80 to 500 ms. The reported Ca^2+^ transients increase linearly over a Δ*F*/*F*_0_ range of 1 to 5 (*n* = 16; see fig. S13), suggesting that the Cal-520 dye fluorescent changes did not saturate over this range [also see (*49*)].

### Modeling

A simple multicompartmental passive “ball and stick” model with number of segments following the d_lambda rule (*50*) and passive properties *R*_a_ = 181 Ω·cm, *C*_m_ = 1 μF/cm^2,^ and a leak conductance = 0.0002 S/cm^2^, which gave an *R*_in_ of 165 MΩ, were adopted from Carnevale and Hines (*50*) and Krueppel *et al.* (*14*). The model is available on GitHub (https://github.com/tonykelly00/DSC_model). A soma (20 μm in diameter) contained one or two dendrites (1 μm in diameter and 250 μm in length). The transfer (*Z*_c_) and input impedance (*Z*_n_) were determined from the model. Simulations were run in the Neuron 7.8 simulation environment.

Synaptic conductances were modeled using point mechanisms for AMPA and NMDA conductances and were placed on the dendrite at 90 and 220 μm from the soma. The AMPA conductance was modeled with a biexponential function (AMPA tau_rise = 0.05 ms and AMPA tau_decay = 2 ms), and the NMDA conductance was modeled with a biexponential function with a voltage-dependent Mg^2+^ block (NMDA tau_rise = 0.33 ms and NMDA tau_decay = 50 ms). The Mg^2+^ block of NMDA conductance was dependent on voltage with a sensitivity of gamma = 0.06 mV^−1^ and on Mg^2+^ concentration with a sensitivity of eta = 0.05 mM^−1^.

The range of unitary synaptic conductances [0.1 to 1 nS; see (*51*)] elicited EPSP amplitudes in the model, which covered the range of somatic EPSP amplitudes that were experimentally measured (0.2 to 2.0 mV; see fig. S10). A unitary synaptic conductance was selected that elicited a uEPSP equivalent to the average uEPSP measured experimentally at the soma. Multiples of the uEPSPs conductance were used to simulate compound EPSPs (measured EPSP) and the amplitude compared to the arithmetic sum of the unitary response (expected EPSP). It should be noted that the relationship between measured and expected EPSP amplitudes was insensitive to the unitary synaptic conductance (gSyn; fig. S8C) over the unitary somatic EPSP amplitudes typically observed experimentally (uEPSP amplitudes of 0.2 to 2 mV; see fig. S10). To assess the synaptic gain, we determined the EPSP ratio as the measured EPSP amplitude relative to the expected EPSP over the range of expected EPSPs amplitudes of 8 to 10 mV. In experiments assessing the interaction of proximal and distal inputs, NMDA recruitment fraction is the fraction of total NMDA receptors recruited. DSCs were modeled as short (1 μm) dendritic segments with a diameter of 0.2 μm, and one to seven DSCs were located between 140 and 200 μm from the soma at 10-μm intervals.

### Realistic compartmental modeling

Three experimentally derived dendritic morphologies were reconstructed from ExM data: two dendrites from cell C1_03 (denoted C1_03_d3 and C1_03_d2) and one from cell C1_02 (C1_02_d2). The primary morphology used for single-cell analyses (fig. S9) is C1_03_d3, with DSC = 5. Each reconstruction was converted to SWC format and imported into the NEURON simulation environment. Dendrites were modeled with a spherical somatic compartment (diameter, 20 μm) and passive membrane properties identical to those used in the ball-and-stick model (axial resistance *R*_a_ = 181 Ω·cm, specific membrane capacitance *C*_m_ = 1 μF/cm^2^, and leak conductance gleak = 0.0002 S/cm^2^). DSC geometry was preserved as in the ball-and-stick model (diameter, 0.33 μm), and DSCs were systematically removed by expanding their diameter to match the average dendritic borders. The number of active DSCs ranged from 0 to the maximum present in each morphology (five for C1_03_d3).

The distal synaptic input site was positioned 50 μm from the dendritic tip (corresponding to a normalized dendritic position of ∼0.80 to 0.90 depending on morphology). The proximal input site was fixed at a normalized position of 0.36 along the total dendritic path length. Somatic and local dendritic voltages, synaptic integration curves, and EPSP ratios were computed as described for the ball-and-stick model, with synaptic gain quantified at a target somatic EPSP amplitude of 10 mV (NMDA/AMPA conductance ratio = 1.1). Active Na^+^ and K^+^ conductances were incorporated and calibrated to reproduce physiologically realistic subthreshold synaptic integration without somatic spiking, following the channel distributions described in Krueppel *et al.* (*14*). To calibrate across morphologies, the synaptic conductance (g*) was determined for each morphology individually by root-finding (Brent’s method) to match the target somatic EPSP of 10 mV at DSC = 0.

### Statistical analysis

Values are presented as means ± SEM. *z*-score transformation was applied to normalize the diameter to the median of branch. Student’s *t* test was used for comparisons between two independent groups when data were normally distributed. Two-way analysis of variance (ANOVA) was performed to assess the interaction effects between two independent variables on a continuous outcome. Information about sample sizes and experimental conditions are given in figure legends.

## References

[1] J. Tønnesen, U. V. Nägerl, Dendritic spines as tunable regulators of synaptic signals. Front. Psych. 7, 101 (2016). 10.3389/fpsyt.2016.00101 PMC4899469 27340393

[2] B. L. Bloodgood, B. L. Sabatini, Ca^2+^ signaling in dendritic spines. Curr. Opin. Neurobiol. 17, 345–351 (2007). 17451936 10.1016/j.conb.2007.04.003

[3] R. Yuste, A. Majewska, K. Holthoff, From form to function: Calcium compartmentalization in dendritic spines. Nat. Neurosci. 3, 653–659 (2000). 10862697 10.1038/76609

[4] R. Yuste, Electrical compartmentalization in dendritic spines. Annu. Rev. Neurosci. 36, 429–449 (2013). 23724997 10.1146/annurev-neuro-062111-150455

[5] V. H. Cornejo, N. Ofer, R. Yuste, Voltage compartmentalization in dendritic spines in vivo. Science 375, 82–86 (2022). 34762487 10.1126/science.abg0501 PMC8942082

[6] P. Poirazi, A. Papoutsi, Illuminating dendritic function with computational models. Nat. Rev. Neurosci. 21, 303–321 (2020). 32393820 10.1038/s41583-020-0301-7

[7] W. Rall, Electrophysiology of a dendritic neuron model. Biophys. J. 2, 145–167 (1962). 14490040 10.1016/s0006-3495(62)86953-7 PMC1366481

[8] N. Spruston, G. Stuart, M. Häusser, “Principles of dendritic integration,” in *Dendrites* (Oxford Univ. Press, ed. 3, 2016), pp. 351–398.

[9] T. Branco, M. Häusser, The single dendritic branch as a fundamental functional unit in the nervous system. Curr. Opin. Neurobiol. 20, 494–502 (2010). 20800473 10.1016/j.conb.2010.07.009

[10] J. Tønnesen, G. Katona, B. Rózsa, U. V. Nägerl, Spine neck plasticity regulates compartmentalization of synapses. Nat. Neurosci. 17, 678–685 (2014). 24657968 10.1038/nn.3682

[11] The MICrONS Consortium, Functional connectomics spanning multiple areas of mouse visual cortex. Nature 640, 435–447 (2025). 40205214 10.1038/s41586-025-08790-w PMC11981939

[12] A. Shapson-Coe, M. Januszewski, D. R. Berger, A. Pope, Y. Wu, T. Blakely, R. L. Schalek, P. H. Li, S. Wang, J. Maitin-Shepard, N. Karlupia, S. Dorkenwald, E. Sjostedt, L. Leavitt, D. Lee, J. Troidl, F. Collman, L. Bailey, A. Fitzmaurice, R. Kar, B. Field, H. Wu, J. Wagner-Carena, D. Aley, J. Lau, Z. Lin, D. Wei, H. Pfister, A. Peleg, V. Jain, J. W. Lichtman, A petavoxel fragment of human cerebral cortex reconstructed at nanoscale resolution. Science 384, eadk4858 (2024). 38723085 10.1126/science.adk4858 PMC11718559

[13] A. Bak, H. Koch, K. M. J. van Loo, K. Schmied, B. Gittel, Y. Weber, J. Ort, N. Schwarz, S. C. Tauber, T. V. Wuttke, D. Delev, Human organotypic brain slice cultures: A detailed and improved protocol for preparation and long-term maintenance. J. Neurosci. Methods 404, 110055 (2024). 38184112 10.1016/j.jneumeth.2023.110055

[14] R. Krueppel, S. Remy, H. Beck, Dendritic integration in hippocampal dentate granule cells. Neuron 71, 512–528 (2011). 21835347 10.1016/j.neuron.2011.05.043

[15] N. L. Desmond, W. B. Levy, Dendritic caliber and the 3/2 power relationship of dentate granule cells. J. Comp. Neurol. 227, 589–596 (1984). 6470225 10.1002/cne.902270410

[16] C. Bromer, T. M. Bartol, J. B. Bowden, D. D. Hubbard, D. C. Hanka, P. V. Gonzalez, M. Kuwajima, J. M. Mendenhall, P. H. Parker, W. C. Abraham, T. J. Sejnowski, K. M. Harris, Long-term potentiation expands information content of hippocampal dentate gyrus synapses. Proc. Natl. Acad. Sci. U.S.A. 115, E2410–E2418 (2018). 29463730 10.1073/pnas.1716189115 PMC5877922

[17] A. W. Grossman, G. M. Aldridge, K. J. Lee, M. K. Zeman, C. S. Jun, H. S. Azam, T. Arii, K. Imoto, W. T. Greenough, I. J. Rhyu, Developmental characteristics of dendritic spines in the dentate gyrus of Fmr1 knockout mice. Brain Res. 1355, 221–227 (2010). 20682298 10.1016/j.brainres.2010.07.090 PMC3433497

[18] M. Trommald, G. Hulleberg, Dimensions and density of dendritic spines from rat dentate granule cells based on reconstructions from serial electron micrographs. J. Comp. Neurol. 377, 15–28 (1997). 8986869 10.1002/(sici)1096-9861(19970106)377:1<15::aid-cne3>3.0.co;2-m

[19] N. L. Turner, T. Macrina, J. A. Bae, R. Yang, A. M. Wilson, C. Schneider-Mizell, K. Lee, R. Lu, J. Wu, A. L. Bodor, A. A. Bleckert, D. Brittain, E. Froudarakis, S. Dorkenwald, F. Collman, N. Kemnitz, D. Ih, W. M. Silversmith, J. Zung, A. Zlateski, I. Tartavull, S. Yu, S. Popovych, S. Mu, W. Wong, C. S. Jordan, M. Castro, J. Buchanan, D. J. Bumbarger, M. Takeno, R. Torres, G. Mahalingam, L. Elabbady, Y. Li, E. Cobos, P. Zhou, S. Suckow, L. Becker, L. Paninski, F. Polleux, J. Reimer, A. S. Tolias, R. C. Reid, N. M. Da Costa, H. S. Seung, Reconstruction of neocortex: Organelles, compartments, cells, circuits, and activity. Cell 185, 1082–1100.e24 (2022). 35216674 10.1016/j.cell.2022.01.023 PMC9337909

[20] W. J. Wright, N. G. Hedrick, T. Komiyama, Distinct synaptic plasticity rules operate across dendritic compartments in vivo during learning. Science 388, 322–328 (2025). 40245144 10.1126/science.ads4706 PMC12906676

[21] R. Yasuda, Biophysics of biochemical signaling in dendritic spines: Implications in synaptic plasticity. Biophys. J. 113, 2152–2159 (2017). 28866426 10.1016/j.bpj.2017.07.029 PMC5700242

[22] Y. Hayashi, A. K. Majewska, Dendritic spine geometry: Functional implication and regulation. Neuron 46, 529–532 (2005). 15944122 10.1016/j.neuron.2005.05.006

[23] M. Sehgal, D. A. Filho, G. Kastellakis, S. Kim, J. Lee, Y. Shen, S. Huang, A. Lavi, G. Fernandes, I. Davila Mejia, S. S. Martin, A. Pekcan, M. S. Wu, W. D. Heo, P. Poirazi, J. T. Trachtenberg, A. J. Silva, Compartmentalized dendritic plasticity in the mouse retrosplenial cortex links contextual memories formed close in time. Nat. Neurosci. 28, 602–615 (2025). 39962274 10.1038/s41593-025-01876-8 PMC11893454

[24] L. Kanari, Y. Shi, A. Arnaudon, N. Barros-Zulaica, R. Benavides-Piccione, J. S. Coggan, J. DeFelipe, K. Hess, H. D. Mansvelder, E. J. Mertens, J. Meystre, R. de Campos Perin, M. Pezzoli, R. T. Daniel, R. Stoop, I. Segev, H. Markram, C. P. J. de Kock, Of mice and men: Dendritic architecture differentiates human from mouse neuronal networks. iScience 28, 112928 (2025). 40687823 10.1016/j.isci.2025.112928 PMC12274884

[25] A. Gidon, T. A. Zolnik, P. Fidzinski, F. Bolduan, A. Papoutsi, P. Poirazi, M. Holtkamp, I. Vida, M. E. Larkum, Dendritic action potentials and computation in human layer 2/3 cortical neurons. Science 367, 83–87 (2020). 31896716 10.1126/science.aax6239

[26] G. Eyal, M. B. Verhoog, G. Testa-Silva, Y. Deitcher, R. Benavides-Piccione, J. DeFelipe, C. P. J. de Kock, H. D. Mansvelder, I. Segev, Human cortical pyramidal neurons: From spines to spikes via models. Front. Cell. Neurosci. 12, 181 (2018). 30008663 10.3389/fncel.2018.00181 PMC6034553

[27] E. D'Este, D. Kamin, F. Göttfert, A. El-Hady, S. W. Hell, STED nanoscopy reveals the ubiquity of subcortical cytoskeleton periodicity in living neurons. Cell Rep. 10, 1246–1251 (2015). 25732815 10.1016/j.celrep.2015.02.007

[28] B. Han, R. Zhou, C. Xia, X. Zhuang, Structural organization of the actin-spectrin-based membrane skeleton in dendrites and soma of neurons. Proc. Natl. Acad. Sci. U.S.A. 114, E6678–E6685 (2017). 28739933 10.1073/pnas.1705043114 PMC5559029

[29] J. Bär, O. Kobler, B. van Bommel, M. Mikhaylova, Periodic F-actin structures shape the neck of dendritic spines. Sci. Rep. 6, 37136 (2016). 27841352 10.1038/srep37136 PMC5107894

[30] M. Arizono, V. V. G. K. Inavalli, A. Panatier, T. Pfeiffer, J. Angibaud, F. Levet, M. J. T. ter Veer, J. Stobart, L. Bellocchio, K. Mikoshiba, G. Marsicano, B. Weber, S. H. R. Oliet, U. V. Nägerl, Structural basis of astrocytic Ca^2+^ signals at tripartite synapses. Nat. Commun. 11, 1906 (2020). 32312988 10.1038/s41467-020-15648-4 PMC7170846

[31] S. Aten, C. M. Kiyoshi, E. P. Arzola, J. A. Patterson, A. T. Taylor, Y. Du, A. M. Guiher, M. Philip, E. G. Camacho, D. Mediratta, K. Collins, K. Boni, S. A. Garcia, R. Kumar, A. N. Drake, A. Hegazi, L. Trank, E. Benson, G. Kidd, D. Terman, M. Zhou, Ultrastructural view of astrocyte arborization, astrocyte-astrocyte and astrocyte-synapse contacts, intracellular vesicle-like structures, and mitochondrial network. Prog. Neurobiol. 213, 102264 (2022). 35283239 10.1016/j.pneurobio.2022.102264 PMC9050955

[32] M. Arizono, U. V. Nägerl, Deciphering the functional nano-anatomy of the tripartite synapse using stimulated emission depletion microscopy. Glia 70, 607–618 (2022). 34664734 10.1002/glia.24103

[33] M. Doengi, A. J. Krupp, N. Körber, V. Stein, SynCAM 1 improves survival of adult-born neurons by accelerating synapse maturation. Hippocampus 26, 319–328 (2016). 26332750 10.1002/hipo.22524

[34] J. Bürgers, I. Pavlova, J. E. Rodriguez-Gatica, C. Henneberger, M. Oeller, J. A. Ruland, J. P. Siebrasse, U. Kubitscheck, M. K. Schwarz, Light-sheet fluorescence expansion microscopy: Fast mapping of neural circuits at super resolution. Neurophotonics 6, 15005 (2019). 10.1117/1.NPh.6.1.015005 PMC6368534 30796881

[35] J. E. Rodriguez-Gatica, V. Iefremova, L. Sokhranyaeva, S. W. C. Au Yeung, Y. Breitkreuz, O. Brüstle, M. K. Schwarz, U. Kubitscheck, Imaging three-dimensional brain organoid architecture from meso- to nanoscale across development. Development 149, dev200439 (2022). 35899577 10.1242/dev.200439

[36] J. Schindelin, I. Arganda-Carreras, E. Frise, V. Kaynig, M. Longair, T. Pietzsch, S. Preibisch, C. Rueden, S. Saalfeld, B. Schmid, J.-Y. Tinevez, D. J. White, V. Hartenstein, K. Eliceiri, P. Tomancak, A. Cardona, Fiji: An open-source platform for biological-image analysis. Nat. Methods 9, 676–682 (2012). 22743772 10.1038/nmeth.2019 PMC3855844

[37] S. Preibisch, S. Saalfeld, P. Tomancak, Globally optimal stitching of tiled 3D microscopic image acquisitions. Bioinformatics 25, 1463–1465 (2009). 19346324 10.1093/bioinformatics/btp184 PMC2682522

[38] P. Bethge, R. Chéreau, E. Avignone, G. Marsicano, U. V. Nägerl, Two-photon excitation STED microscopy in two colors in acute brain slices. Biophys. J. 104, 778–785 (2013). 23442956 10.1016/j.bpj.2012.12.054 PMC3576543

[39] M. J. T. ter Veer, T. Pfeiffer, U. V. Nägerl, Two-photon STED microscopy for nanoscale imaging of neural morphology in vivo. Methods Mol. Biol. 1663, 45–64 (2017). 28924658 10.1007/978-1-4939-7265-4_5

[40] C. M. Godale, E. V. Parkins, C. Gross, S. C. Danzer, Impact of raptor and Rictor deletion on hippocampal pathology following status epilepticus. J. Mol. Neurosci. 72, 1243–1258 (2022). 35618880 10.1007/s12031-022-02030-w PMC9571976

[41] C. M. Godale, S. Yaser, A. W. Drake, K. L. Kraus, N. Mayer, M. R. Dusing, C. L. LaSarge, C. Gross, S. C. Danzer, Cell-intrinsic regulation of epilepsy-associated pathology by mTORC1 and mTORC2. J. Neurosci. 46, e1211252026 (2026). 41916759 10.1523/JNEUROSCI.1211-25.2026 PMC13113615

[42] J. R. Kremer, D. N. Mastronarde, J. R. McIntosh, Computer visualization of three-dimensional image data using IMOD. J. Struct. Biol. 116, 71–76 (1996). 8742726 10.1006/jsbi.1996.0013

[43] M. Abdellah, J. J. G. Cantero, N. R. Guerrero, A. Foni, J. S. Coggan, C. Calì, M. Agus, E. Zisis, D. Keller, M. Hadwiger, P. J. Magistretti, H. Markram, F. Schürmann, Ultraliser: A framework for creating multiscale, high-fidelity and geometrically realistic 3D models for in silico neuroscience. Brief. Bioinform. 24, bbac491 (2023). 36434788 10.1093/bib/bbac491 PMC9851302

[44] K. D. Schünemann, R. M. Hattingh, M. B. Verhoog, D. Yang, A. V. Bak, S. Peter, K. M. J. van Loo, S. Wolking, D. Kronenberg-Versteeg, Y. Weber, N. Schwarz, J. V. Raimondo, R. Melvill, S. A. Tromp, J. T. Butler, A. Höllig, D. Delev, T. V. Wuttke, B. M. Kampa, H. Koch, Comprehensive analysis of human dendritic spine morphology and density. J. Neurophysiol. 133, 1086–1102 (2025). 40013734 10.1152/jn.00622.2024

[45] I. Arganda-Carreras, R. Fernández-González, A. Muñoz-Barrutia, C. Ortiz-De-Solorzano, 3D reconstruction of histological sections: Application to mammary gland tissue. Microsc. Res. Tech. 73, 1019–1029 (2010). 20232465 10.1002/jemt.20829

[46] J. Ollion, J. Cochennec, F. Loll, C. Escudé, T. Boudier, TANGO: A generic tool for high-throughput 3D image analysis for studying nuclear organization. Bioinformatics 29, 1840–1841 (2013). 23681123 10.1093/bioinformatics/btt276 PMC3702251

[47] C. Schmidt-Hieber, P. Jonas, J. Bischofberger, Enhanced synaptic plasticity in newly generated granule cells of the adult hippocampus. Nature 429, 184–187 (2004). 15107864 10.1038/nature02553

[48] N. Ji, J. C. Magee, E. Betzig, High-speed, low-photodamage nonlinear imaging using passive pulse splitters. Nat. Methods 5, 197–202 (2008). 18204458 10.1038/nmeth.1175

[49] M. Tada, A. Takeuchi, M. Hashizume, K. Kitamura, M. Kano, A highly sensitive fluorescent indicator dye for calcium imaging of neural activity in vitro and in vivo. Eur. J. Neurosci. 39, 1720–1728 (2014). 24405482 10.1111/ejn.12476 PMC4232931

[50] N. T. Carnevale, M. L. Hines, *The NEURON Book* (Cambridge Univ. Press, 2010).

[51] S. R. Williams, S. J. Mitchell, Direct measurement of somatic voltage clamp errors in central neurons. Nat. Neurosci. 11, 790–798 (2008). 18552844 10.1038/nn.2137

