# Supplementary material for: Dendritic shaft constrictions shape synaptic integration in neurons
Source: Sci Adv. 2026 Sep 11;12(37):eaec4911. doi: 10.1126/sciadv.aec4911 (PMC13564900; doi:10.1126/sciadv.aec4911)
Supplement: Supplementary file 1 [file sciadv.aec4911_sm.pdf]

Science Advances
AAAS

Supplementary Materials for

Dendritic shaft constrictions shape synaptic integration in neurons

Tony Kelly *et al.*

Corresponding author: Tony Kelly, tony.kelly00@gmail.com; Heinz Beck, heinz.beck@ukbonn.de

Sci. Adv. **12**, eaec4911 (2026)
DOI: 10.1126/sciadv.aec4911

This PDF file includes:

Figs. S1 to S13

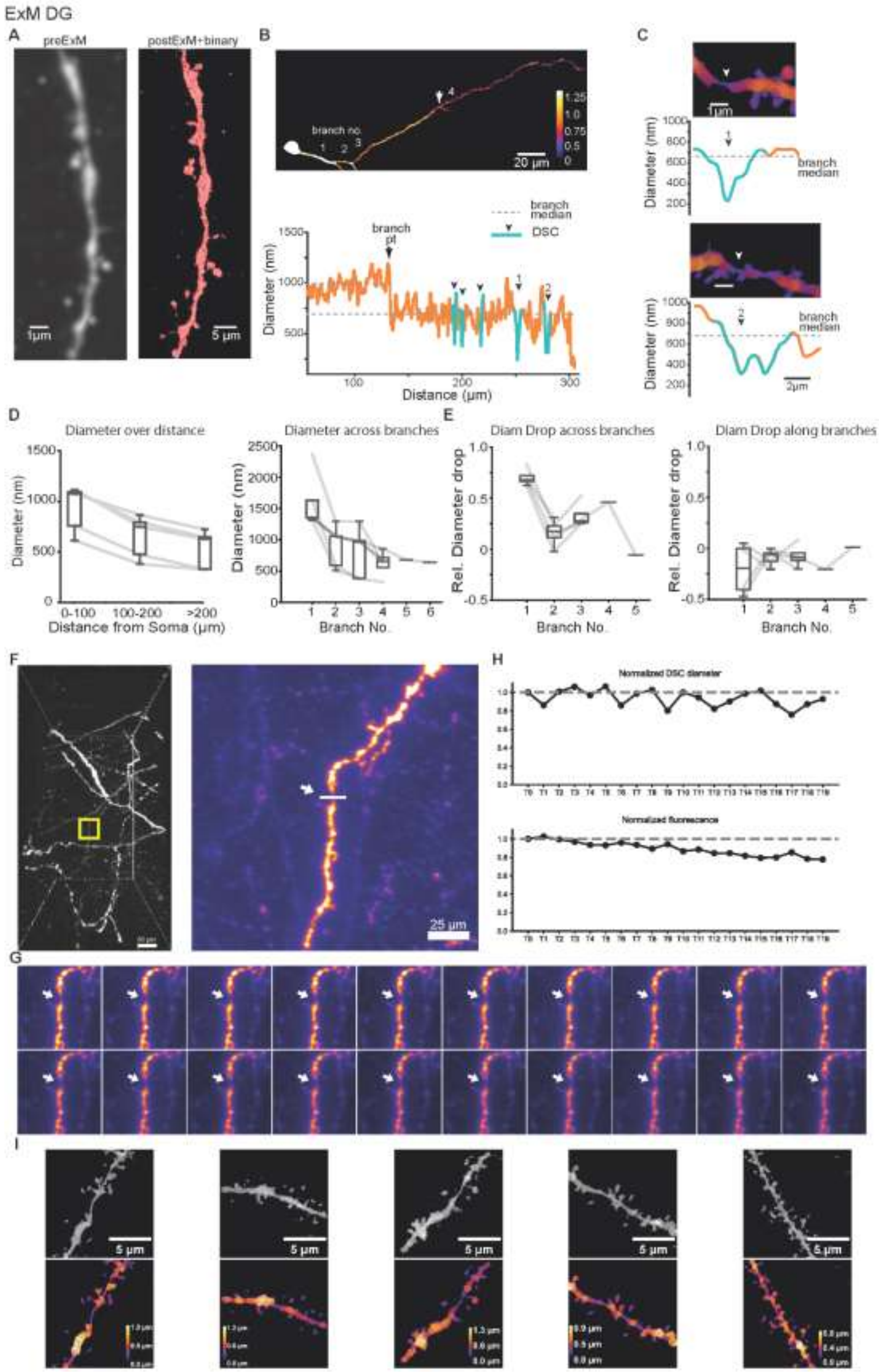
ExM DG
A
preExM
postExM+binary
1µm
5 µm
B
branch no.
1 2 3
4
20 µm
1.25
1.0
0.75
0.5
0
branch pt
branch median
DSC
Diameter (nm)
Distance (µm)
C
1µm
Diameter (nm)
branch median
2µm
D
Diameter over distance
Diameter (nm)
Distance from Soma (µm)
0-100
100-200
>200
Diameter across branches
Branch No.
E
Diam Drop across branches
Rel. Diameter drop
Branch No.
Diam Drop along branches
F
25 µm
H
Normalized DSC diameter
Normalized fluorescence
G
I
5 µm

Supplementary Fig. S1: Imaging granule cell dendrites at nano-scale resolution. **A**, left: confocal maximum projection image of granule cell dendritic segment prior to expansion (preExM); right: maximum projection image with overlapping binary image (red) of same segment following ExM. Comparing preExM and postExM images was used to determine the expansion factor of each section. **B**, Example of a 3D reconstruction of an entire individual dendrite from soma to distal tip. Granule cell was sparsely labelled with eGFP using Molony virus transduction. Pseudo-colored with the diameter of the largest non-overlapping sphere (*above*) and plot of diameter against distance from soma for the distal half of the dendrite containing DSCs (*below*). At branch points (arrow) diameters drop as in this example, and remain at similar levels more distally. Drops in diameter by factor of >2 which re-increase to at least 75% were identified as DSCs indicated with cyan lines and arrowheads. Scale is corrected for using the expansion factor. **C**, Example DSCs from B pseudo-colored with the diameter. DSCs indicated with arrows. Scale bar is corrected for the expansion factor. **D,** plots of average shaft diameter binned by distance from soma or Branch No. **E**, Plots showing drop in diameter across branches determined as mean diameter at last 10 µm of parent branch compared to beginning 10 µm of daughter branch and drop in diameter along a branch determined as mean diameter at beginning 10 µm compared to last 10 µm. Drops in diameter expressed as proportions of total drop in diameter over entire dendrite. **F-H,** To assess photodamage, repeated light sheet imaging was performed on expanded tissue containing GFP-labelled and immunostained neurons in a coronal mouse hippocampal slice. Panels show an overview (**F**), and images derived from 20 sequential volume scans (**G**). The images show a clear DSC that seems unchanged over time. DSC diameters also proved to be quantitatively unchanged (panel **H**, top) even though photobleaching was observed (panel **H**, bottom). **I,** Representative examples of four DSCs imaged with STED microscopy.

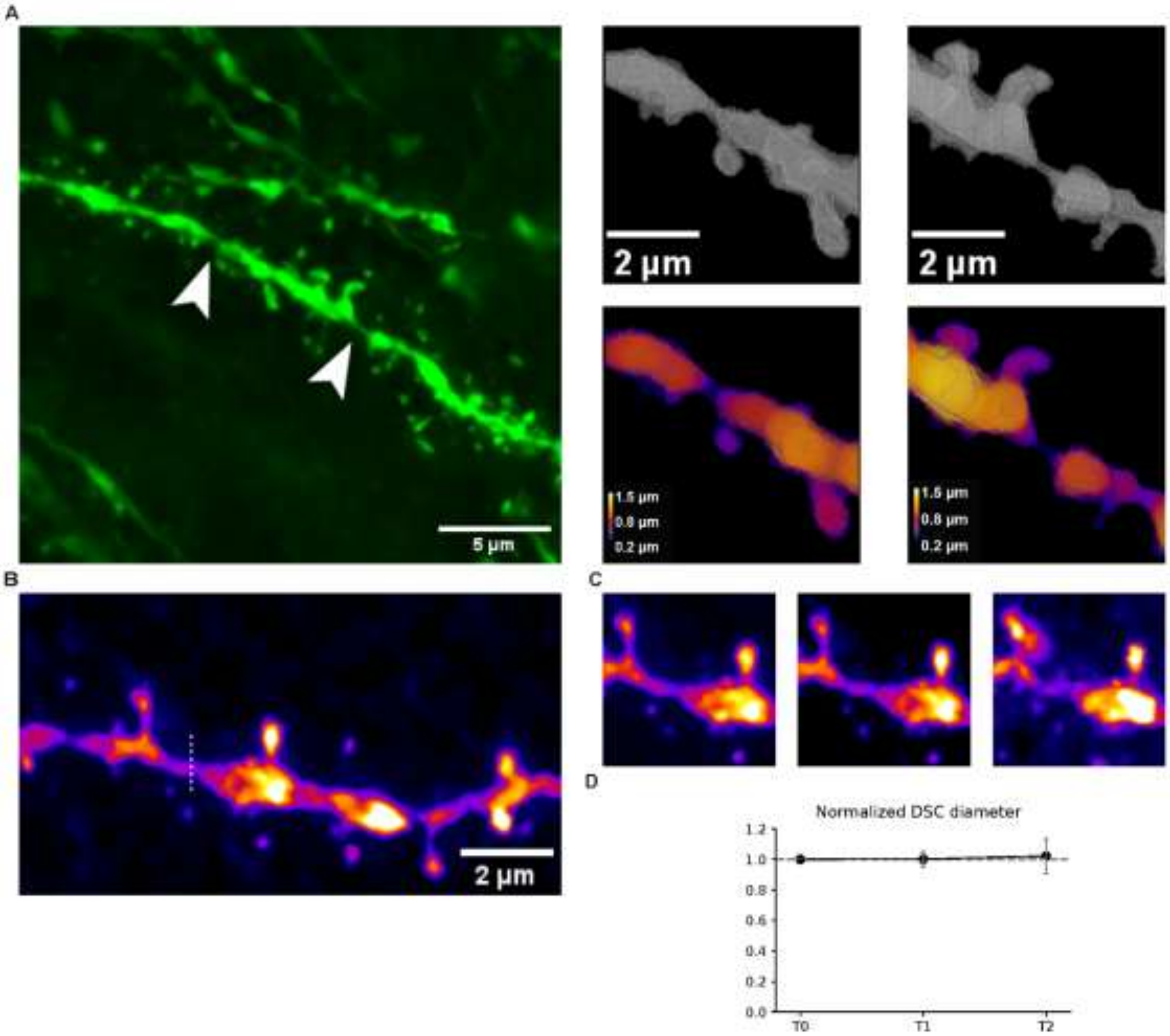

A
5 µm
2 µm
2 µm
1.5 µm
0.8 µm
0.2 µm
1.5 µm
0.8 µm
0.2 µm
B
2 µm
C
D
Normalized DSC diameter
1.2
1.0
0.8
0.6
0.4
0.2
0.0
T0
T1
T2

Supplementary Fig. S2: DSCs detected using image scanning microscopy (ISM). A, representative example of a dendrite imaged with a Nikon NSPARC ISM system (upper panel). Lower panels show two examples of 3D reconstructions of dendritic segments containing DSCs as grey scale image (*left*) or pseudo-colored with the diameter of the largest non-overlapping sphere (*right*). **B-D,** Serial imaging of DSCs with the NSPARC system did not reveal photodamage associated changes in DSC dimensions (stack scan starts on average 41 minutes apart). Images show overview (panel B), and an example of a sequentially imaged stretch of dendrite with a DSC (panel C). Quantitative analysis did not show changes in DSC diameter with repetitive volume scanning (panel D, top), even though photobleaching was observed (panel D, bottom).

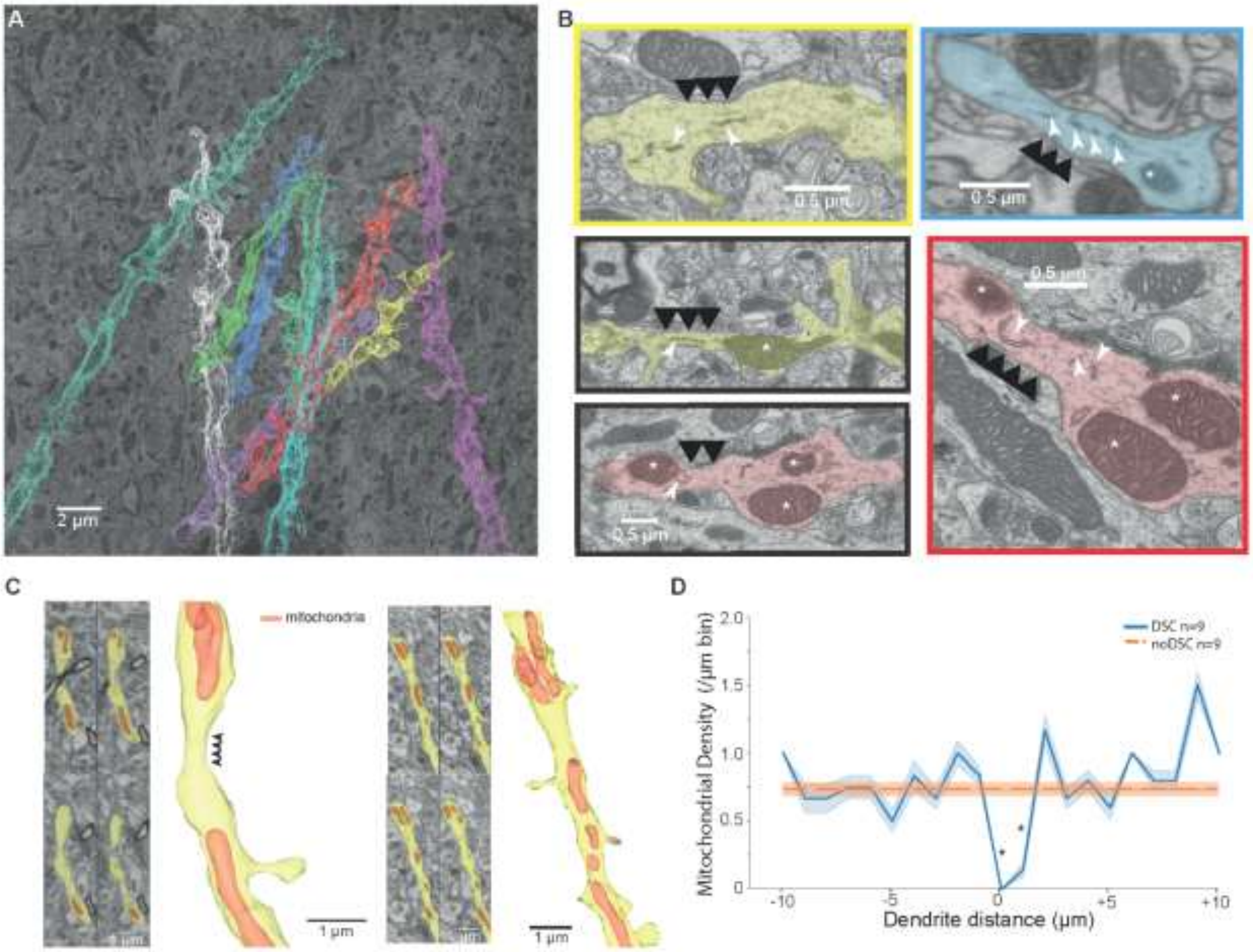
A
2 µm
B
0.5 µm
0.5 µm
0.5 µm
0.5 µm
C
mitochondria
1 µm
1 µm
D
Mitochondrial Density (/µm bin)
Dendrite distance (µm)
DSC n=9
noDSC n=9
2.0
1.5
1.0
0.5
0
-10
-5
0
+5
+10

Supplementary Fig. S3: EM analysis of DSCs, and presence of mitochondria. A, Transmission electron microscope image with overlay of segmented dendrites taken from serial imaging of 50 nm thick sections. **B**, example electron micrographs from 2 different stacks of the outer molecular layer (193 and 160 μm from the granule cell layer). Black arrowheads indicate DSCs where the diameter dropped by >2 compared to the neighboring dendritic shaft. White arrowheads indicate smooth tubular structures and white asterisks indicate mitochondria. **C**, *left*, example micrographs and 3d reconstructions of dendrites showing the absence of mitochondria at DSCs. DSC indicated with arrows. *Right*, also showing dendritic subsegments without mitochondria and without a DSC. **D**, the average mitochondrial density (mean ± sem) in 1 μm bins along dendritic segments containing DSCs, compared to the average mitochondrial density along the entire dendritic segment from all dendritic segments devoid of DSCs. Dendritic segments containing a DSC are aligned with the DSC at 0 μm. The mitochondrial probability was significantly reduced at the site of the DSC compared to the average mitochondrial density from all dendritic segments devoid of DSCs (*, indicates $p<0.05$, Dunnett's test). However, the mere absence of mitochondria was not sufficient for a DSC, as most dendritic subsegments without mitochondria did not exhibit a DSC (14/23, length of 1.7±0.2 μm, n=14). Three DSCs had adjacent mitochondria on both sides (within 1 μm of the DSC). One DSC had no adjacent mitochondria, 4 DSCs displayed mitochondria only on one side and not the other. There was no obvious correspondence of caliber jumps at the edges of DSCs with the presence or absence of mitochondria.

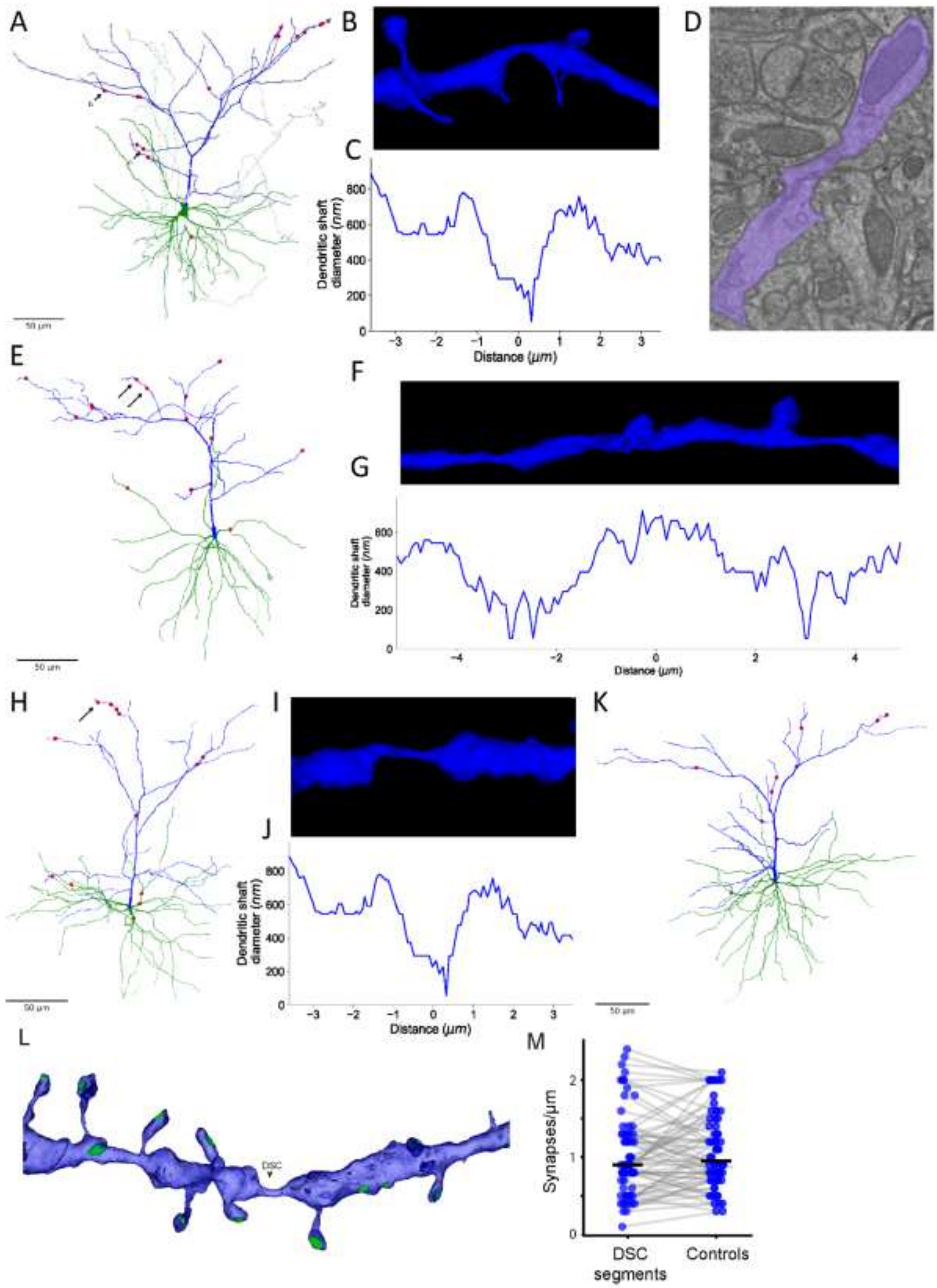

A
B
C
D
E
F
G
H
I
J
K
L
M
50 μm
Dendritic shaft diameter (nm)
Distance (μm)
DSC
Synapses/μm
DSC segments
Controls

Supplementary Fig. S4: DSCs of mouse cortical layer 2/3 pyramidal neurons from serial section EM. A, E, H, K, example reconstructions of pyramidal neurons with locations of DSCs indicated with red circles. **B, F, I,** example reconstructions showing DSCs, **D**, EM photomicrograph of DSC and **C, G, J,** corresponding diameter plots. **L**, example reconstruction of a dendrite with excitatory synapses indicated in green. In rare cases (3/68), a spine emanated from the DSC. **M**, summary plot showing the synapse density in the vicinity (≤5µm from a DSC) and neighboring segments of dendrite 15 µm from the DSC (paired Wilcoxon signed-rank test, $p = 0.31$).

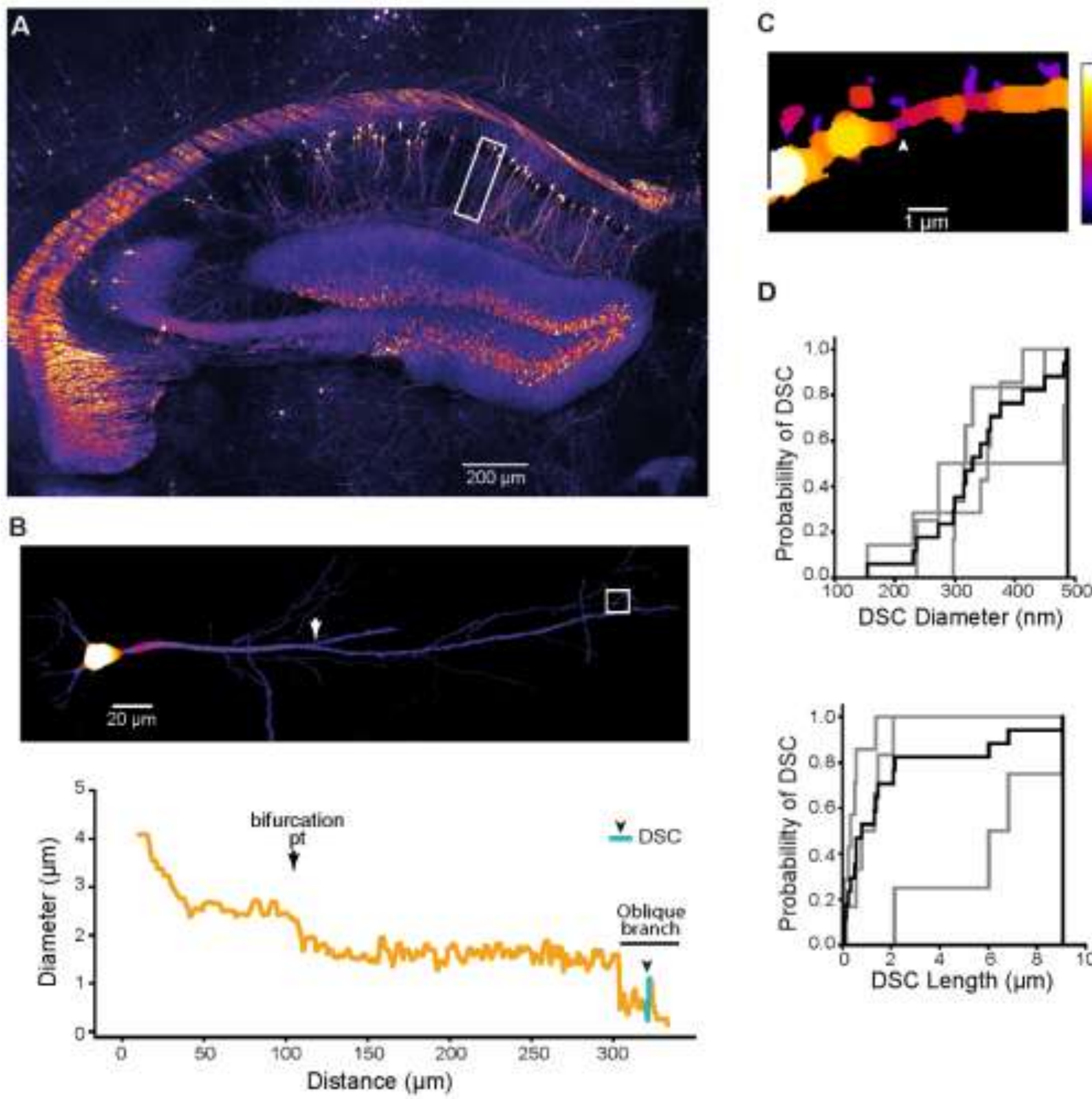

A
200 µm
B
20 µm
bifurcation pt
DSC
Oblique branch
Diameter (µm)
Distance (µm)
C
1 µm
1.0
0.5
0
D
Probability of DSC
DSC Diameter (nm)
Probability of DSC
DSC Length (µm)

Supplementary Fig. S5: Expansion microscopy of CA1 pyramidal cell dendrites. **A**, Maximum projection image of expanded hippocampus acquired using light-sheet microscopy, scale bar corrected for expansion. White box outlines cell shown in B. **B**, Example of a 3d reconstruction of a CA1 pyramidal cell pseudo-colored image, white box indicates oblique branch (above), and plot of diameter against distance from soma (below). **C**, Example DSC from a CA1 pyramidal cell radial oblique dendrite. **D**, CA1 DSCs had an average diameter ± SD of 0.3±0.09 µm (range 0.15-0.48 µm, n=17 DSCs in 3 radial oblique dendrites) and an average length ± SD of 1.7±1.4 µm (range 0.5-9 µm, n=17 DSCs in 3 dendrites).

A

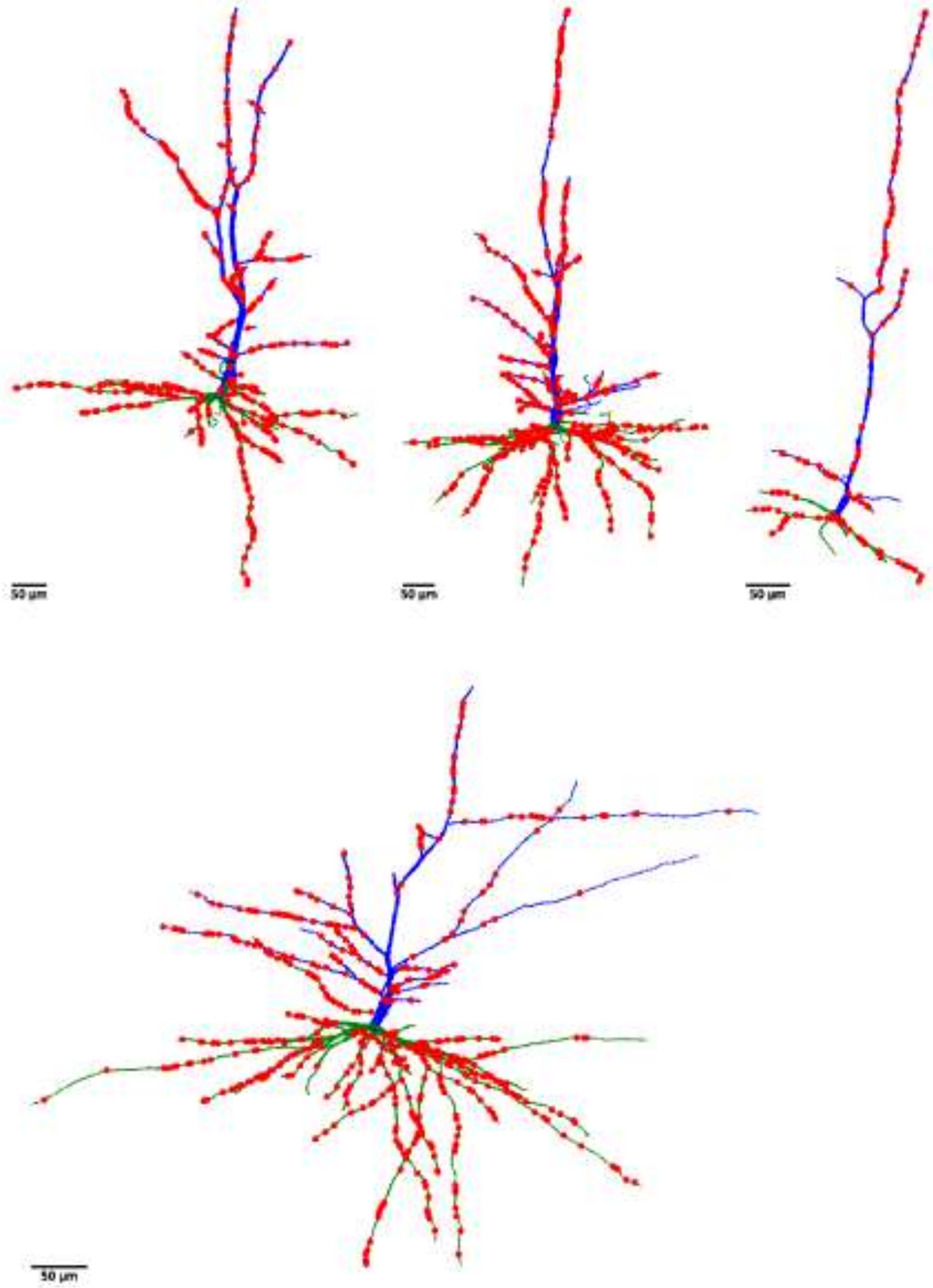

Supplementary Fig. S6: Example reconstructions of human cortical layer 2/3 pyramidal neurons. A, example reconstructions of four different pyramidal neurons from a human serial EM dataset, with locations of DSCs indicated with red circles.

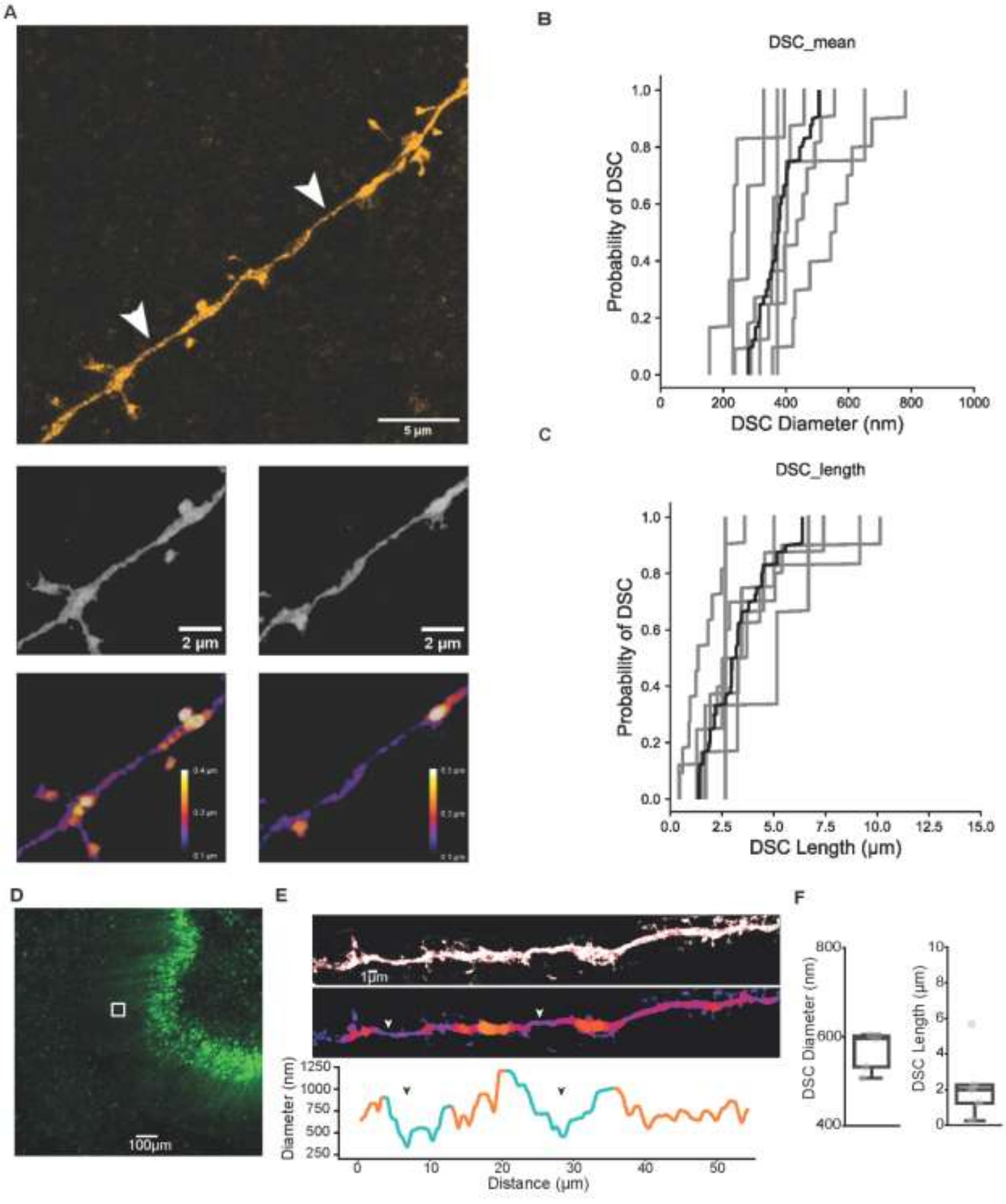

A
5 µm
2 µm
2 µm
B
DSC_mean
Probability of DSC
DSC Diameter (nm)
C
DSC_length
Probability of DSC
DSC Length (µm)
D
100µm
E
1µm
Diameter (nm)
Distance (µm)
F
DSC Diameter (nm)
DSC Length (µm)

Supplementary Fig. S7: DSCs in cortical neurons from acute cortical slice preparations, and human granule cells from hippocampal organotypic slices. A, representative example of a dendrite of a human cortical layer II/III pyramidal neuron from an epilepsy surgical specimen. Cell was recorded in the slice preparation using patch-clamp approaches, filled with biocytin and stained. Magnified inset shows a representative DSC. **B, C,** DSC diameters (B) and lengths (C) derived in this preparation. **D,** confocal maximum projection image of human hippocampal organotypic slice (15 DIV) with dentate granule cells viral transduced with GFP. **E**, granule cell dendrite from D indicated with white box (155 µm from the granule cell layer) reconstructed, binarized and diameter plotted below. DSCs where diameter dropped by >2 indicated by white arrow and highlighted in cyan. **F**, Summary plots of DSC diameter and length for 5 identified DSCs.

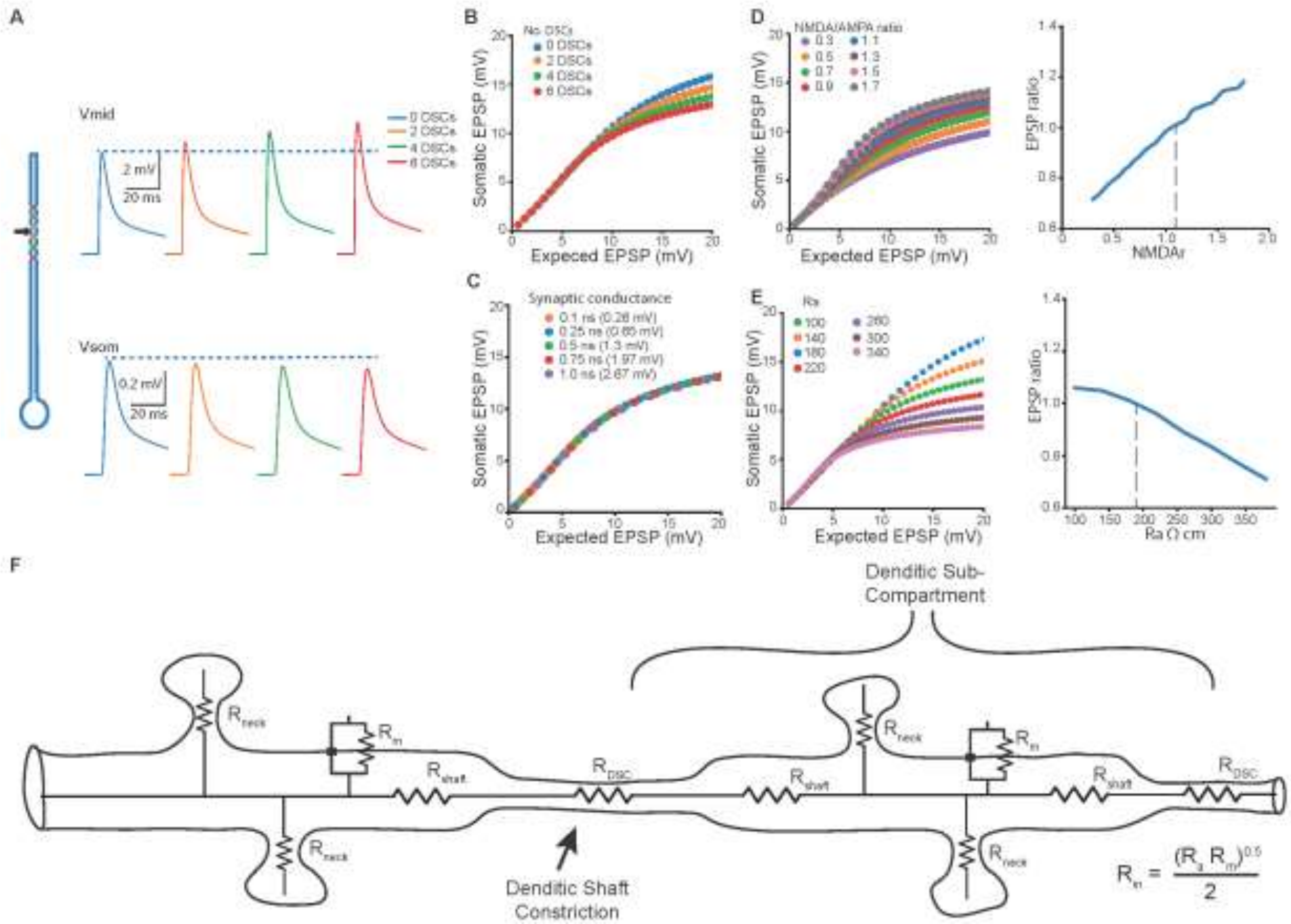

A
Vmid
2 mV
20 ms
0 DSCs
2 DSCs
4 DSCs
6 DSCs
Vsom
0.2 mV
20 ms
B
No DSCs
Somatic EPSP (mV)
Expeced EPSP (mV)
C
Synaptic conductance
0.1 ns (0.26 mV)
0.25 ns (0.65 mV)
0.5 ns (1.3 mV)
0.75 ns (1.97 mV)
1.0 ns (2.67 mV)
Expected EPSP (mV)
D
NMDA/AMPA ratio
Expected EPSP (mV)
EPSP ratio
NMDAr
E
Ra
Ra Ω cm
F
Denditic Sub-
Compartment
Dendritic Shaft
Constriction

Supplementary Fig. S8: Effect of DSC position and neuronal parameters on dendritic integration. **A**, *left*, Schematic representation of 'ball & stick' model consisting of a soma (diameter 20 µm) and dendrite (length 250 µm diameter 1 µm), and showing position of synaptic input (black; 165 µm from the soma) in the dendritic compartment and position of DSCs. *Right*, voltage traces measured at the indicated location in response to synaptic input (synaptic conductance g_{Max}=0.26 nS). The local EPSP amplitude within the subsegment increased (amplitude increases by 8 to 28% when bordered on each side by 2 DSCs (1 on each side), 4 DSCs (2 each side), or 6 DSCs (3 each side), respectively and the efficiency of voltage propagation to the soma decreased (somatic EPSP amplitude decreased by 2 to 7% when bordered by on each side by 2 to 6 DSCs, respectively) **B**, Plots of somatic EPSP amplitude against calculated EPSP amplitude for synaptic input compartmentalised within DSCs (2,4,6 DSCs) or in the absence of DSCs. **C-E,** Effects of synaptic conductance (C), NMDA/AMPA ratio (D), and axial resistance (E) on the relationship between somatic EPSPs and expected EPSP amplitudes in the absence of any DSCs. **C,** Plots of somatic EPSP amplitude against expected EPSP amplitude showing that changing g_{Syn} does not change this relationship in the absence of DSCs. **D,** *Left panel:* Impact of large changes in NMDA/AMPA ratio (indicated in different colors, see legend) on the relationship of somatic EPSP amplitude to expected EPSP amplitude. *left panel*, a NMDA/AMPA ratio of 1.1 (see *3*) was decreased or increased and resulted in increases and decreases in synaptic gain. Synaptic gain determined as mean EPSP ratio of measured vs calculated EPSP amplitudes over the 8-10 mV range of expected EPSPs. *Right panel:* Plot of relationship between the NMDA/AMPA ratio and synaptic gain. Previously reported 1.1 value indicated by dashed line. **E,** *Left panel:* Impact of changes in axial resistance (R_a) on relationship between somatic EPSP amplitude vs. expected EPSP amplitude. *Right panel*, plot of the relationship between R_a and synaptic gain. R_a of 180 Ω cm indicated as dashed line. **F**, Schematic of dendritic segment with dendritic sub-compartment boarded by DSCs. The input impedance or electrical resistance of a compartment depends not only on the membrane resistance but also on the ease by which current flows into the compartment ie the input resistance (R_{in}) of the sub-compartment is related to the membrane (R_m) and axial resistances (R_{shaft} + R_{DSC}) of the compartment. Reductions in diameter increase R_{in} due to increases in both R_m (Ω•cm) and R_a (Ω/cm). R_a is more dramatically increased due to the squared relationship of axial resistance to diameter ($R_a = 4R_A/\pi \bullet d^2$; $R_m = R_M/\pi \bullet d$; where R_M and R_A are the specific membrane (Ω•cm^2) and axial (Ω•cm) resistances and d is diameter)).

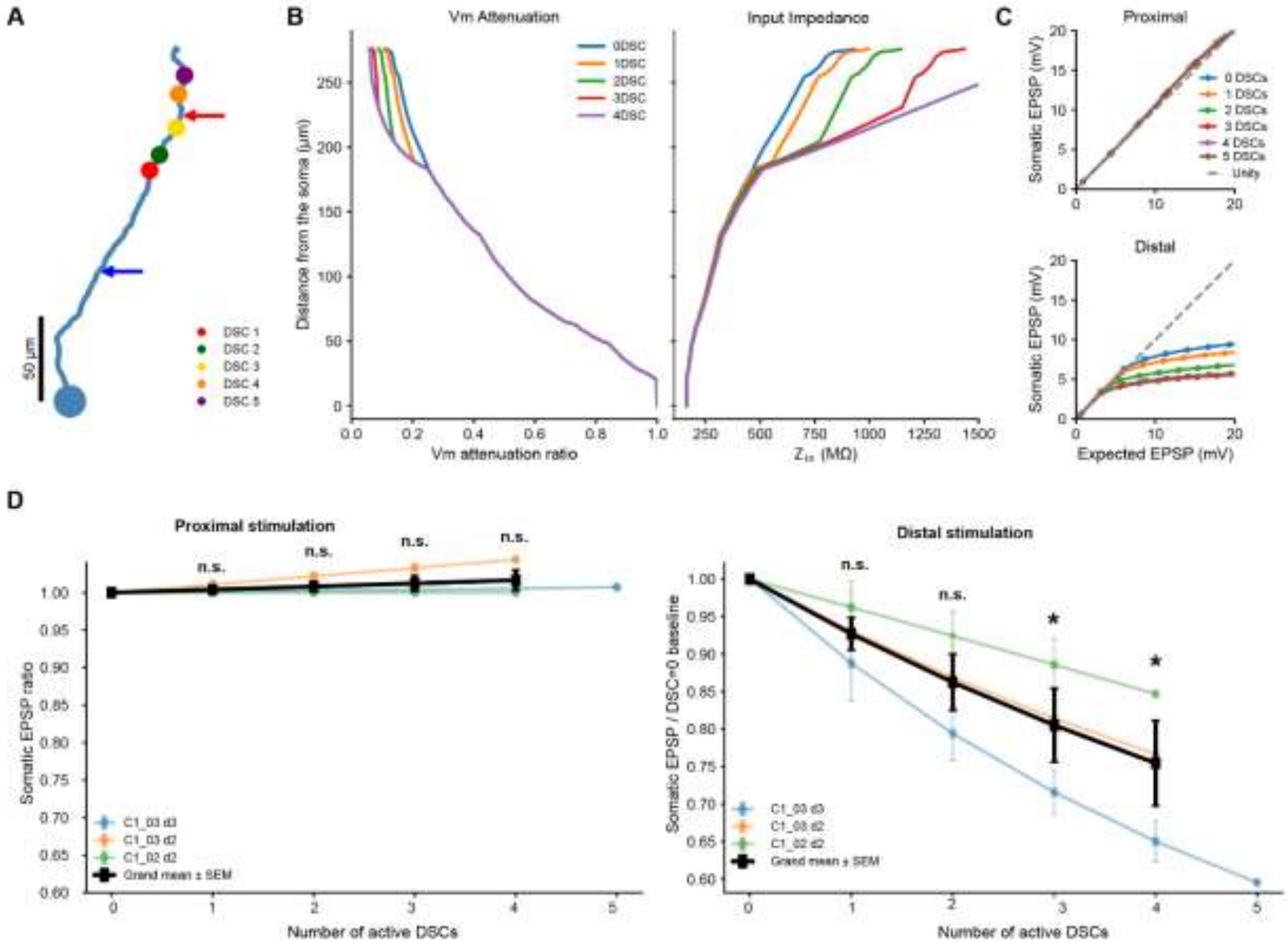

A
B
C
D
Vm Attenuation
Input Impedance
Proximal
Distal
Distance from the soma (μm)
Vm attenuation ratio
Z_in (MΩ)
Somatic EPSP (mV)
Expected EPSP (mV)
0DSC
1DSC
2DSC
3DSC
4DSC
0 DSCs
1 DSCs
2 DSCs
3 DSCs
4 DSCs
5 DSCs
Unity
DSC 1
DSC 2
DSC 3
DSC 4
DSC 5
50 μm
Proximal stimulation
Distal stimulation
Somatic EPSP ratio
Somatic EPSP / DSC=0 baseline
Number of active DSCs
n.s.
*
C1_03 d3
C1_03 d2
C1_02 d2
Grand mean ± SEM

Supplementary Fig. S9: Modeling using realistic morphologies derived from expansion microscopy. A, Morphological reconstruction of a representative granule cell showing the location of five DSC regions (colored circles: DSC 1, red; DSC 2, dark green; DSC 3, gold; DSC 4, dark orange; DSC 5, purple). Arrows indicate the positions of proximal (blue) and distal (red; 50 µm from the dendritic tip) synaptic input sites. **B,** *Left,* Vm attenuation ratio (local-to-somatic voltage) plotted against distance from the soma (µm) for increasing numbers of DSCs (0–4 DSCs; coloured lines). *Right*, input impedance (Z_in, MΩ) plotted against distance from the soma for the same DSC conditions. Progressive addition of DSCs increases both local input impedance and voltage attenuation in the distal dendritic compartment. **C,** Synaptic integration curves showing measured somatic EPSP amplitude (mV) plotted against the expected (linear) EPSP amplitude (mV) for proximal (top) and distal (bottom) synaptic input, across 0–5 DSC conditions (coloured lines). A dashed diagonal line indicates unity. Supralinear integration at distal but not proximal input sites emerges with increasing DSC number. **D,** Pooled combinatorial analysis across three reconstructed morphologies (ExM reconstruction datasets C1_03 d3, C1_03 d2, C1_02 d2; coloured lines). *Left*, somatic EPSP amplitude normalised to the DSC=0 baseline plotted against the number of simultaneously active DSCs for proximal stimulation. *Right*, same for distal stimulation. Black squares and lines indicate grand mean ± SEM across morphologies. Statistical comparisons between DSC=0 and each k>0 condition performed using Mann-Whitney U tests (two-sided) on absolute somatic EPSP amplitudes pooled across morphologies and combinations of DSCs; *$p < 0.05$, **$p < 0.01$, ***$p < 0.001$, n.s. not significant.

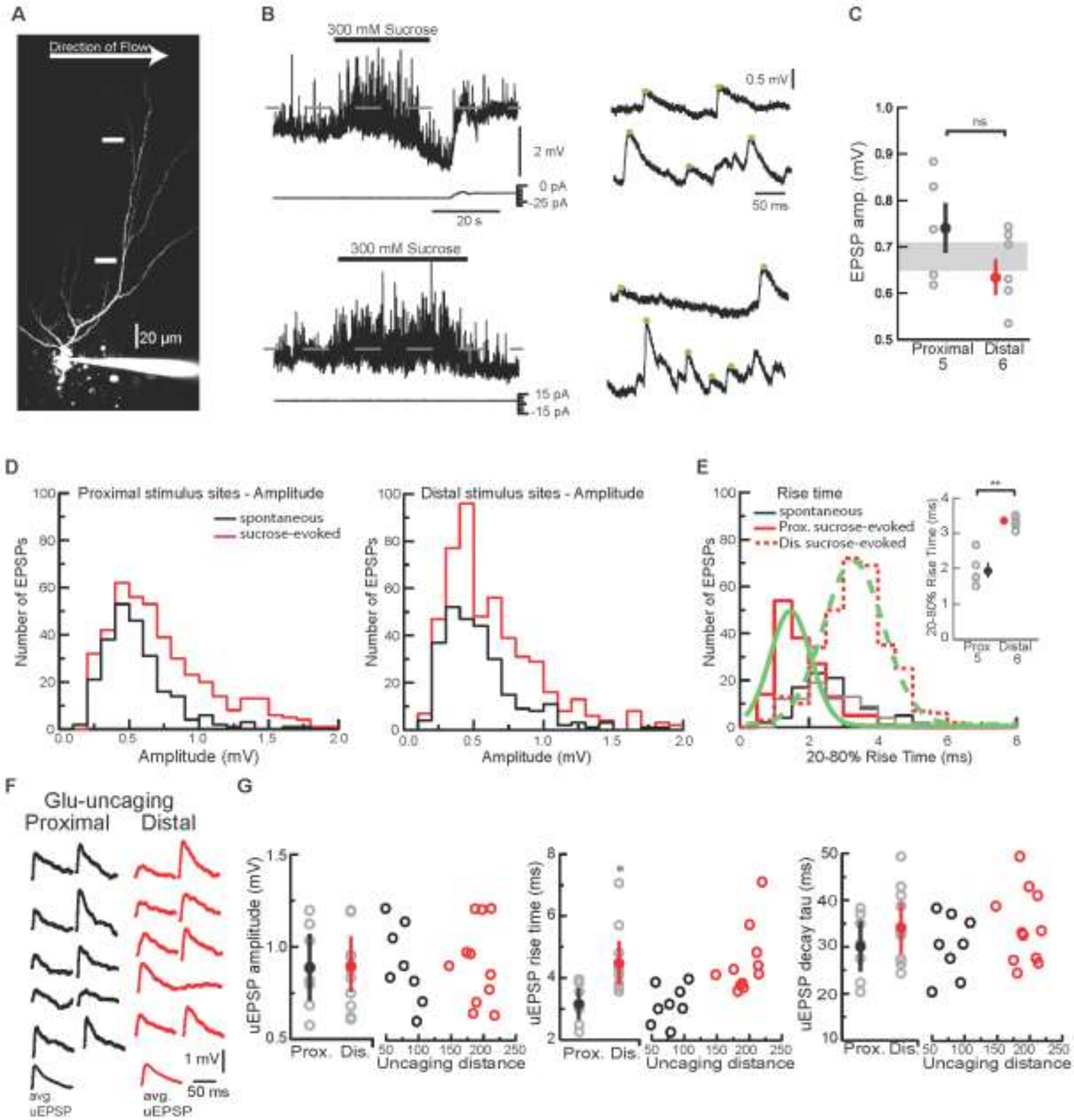

A
Direction of Flow
20 µm
B
300 mM Sucrose
2 mV
0 pA
-25 pA
20 s
0.5 mV
50 ms
15 pA
-15 pA
C
EPSP amp. (mV)
ns
Proximal
5
Distal
6
D
Proximal stimulus sites - Amplitude
spontaneous
sucrose-evoked
Number of EPSPs
Amplitude (mV)
Distal stimulus sites - Amplitude
E
Rise time
spontaneous
Prox. sucrose-evoked
Dis. sucrose-evoked
20-80% Rise Time (ms)
Prox
Dist
F
Glu-uncaging
Proximal
Distal
1 mV
50 ms
avg. uEPSP
G
uEPSP amplitude (mV)
uEPSP rise time (ms)
uEPSP decay tau (ms)
Prox.
Dis.
Uncaging distance

Supplementary Fig. S10: Unitary EPSP properties elicited by sucrose or glutamate uncaging at proximal and distal input sites. **A**, Two-photon maximum projection fluorescent image of granule cell filled with Alexa 594 via a patch-pipette. White lines indicate positions of sucrose pipette. **B**, Sucrose-evoked EPSPs at proximal and distal sites. **C,** mean amplitudes of sucrose-evoked EPSPs were similar at proximal and distal sites (mean ± sem; Student's t-test $p=0.1$). Grey shaded area indicates the mean(±sem) of 0.68±0.03 across all input sites and was used for computational model. **D**, distribution of sucrose-evoked EPSPs amplitudes at two inputs site. **E**, rise-time of sucrose-evoked EPSPs differed between proximal and distal input sites inset shows mean rise times for input sites (Student's t-test $p<0.01$). **F**, single spine EPSPs evoked by glutamate uncaging at distal (red) and proximal (black) input sites. **G**, properties of individual single spine responses. The uEPSPs exhibited slower rise times at the distal site, as expected (3.2±0.5 ms vs. 4.5±0.7 ms, n=8 vs 11, for proximal and distal sites respectively; $p<0.01$ Student's t-test). Between proximal and distal sites there was no significant difference in the uEPSP amplitudes (0.89±0.18 mV vs 0.90±0.15 mV, n=8 and 11; Student's t-test $p=0.94$) or decay time constants (30.2±5.5 ms vs. 34±5.4 ms, n=8 vs 11, for proximal and distal sites respectively; $p=0.22$ Student's t-test).

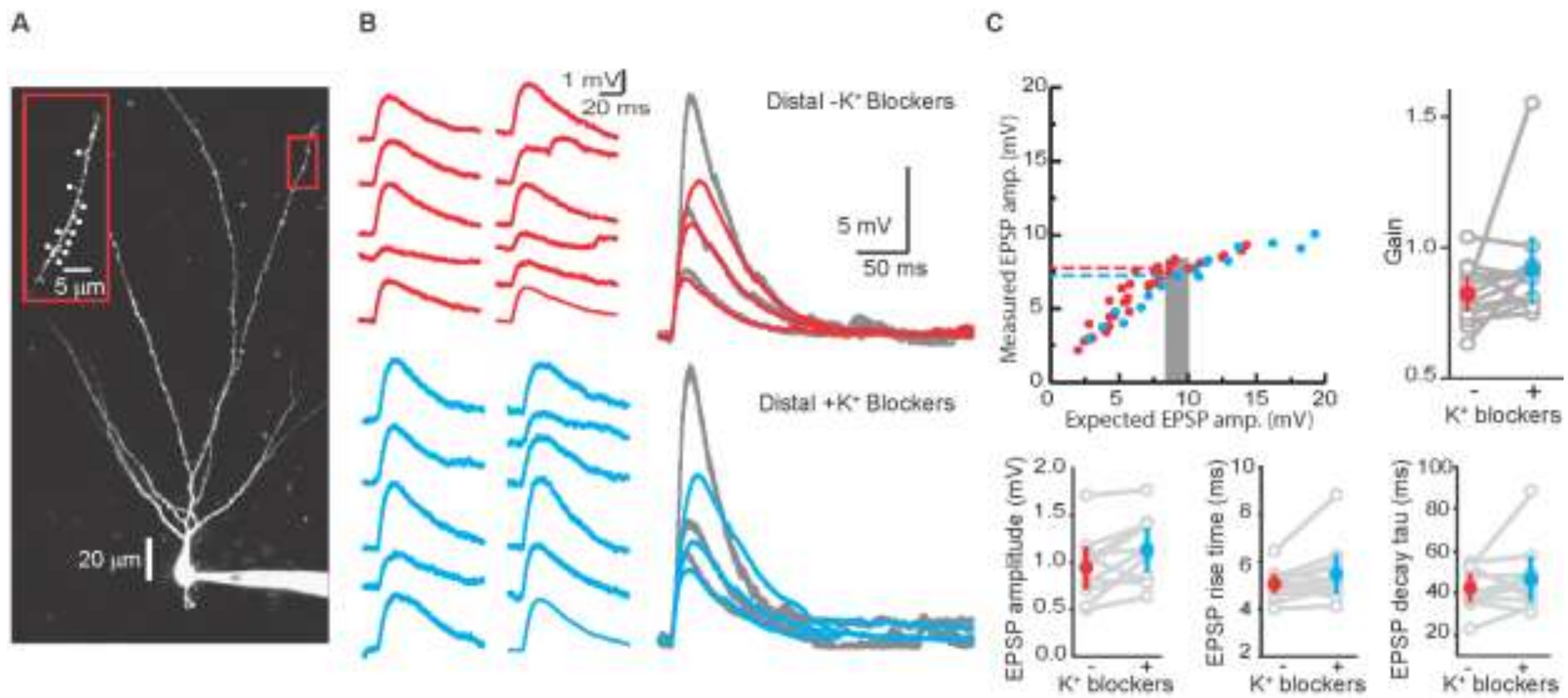

A
5 µm
20 µm
B
1 mV
20 ms
Distal -K⁺ Blockers
5 mV
50 ms
Distal +K⁺ Blockers
C
Measured EPSP amp. (mV)
Expected EPSP amp. (mV)
Gain
K⁺ blockers
EPSP amplitude (mV)
EPSP rise time (ms)
EPSP decay tau (ms)

Supplementary Fig. S11: Involvement of voltage-gated and synaptic conductances. A, Two photon image of granule cell filled with Alexa 594. Two-photon glutamate uncaging was performed at distal (red box) input sites. *Insets*: higher magnification images with uncaging points marked by dots. **B**, *left*, single spine EPSPs evoked by glutamate uncaging at distal input sites in the absence (red) and presence (blue) of K^+ channel blockers (1 mM TEA and 200 μM 4-AP). *Right*, compound EPSPs evoked by near-synchronous glutamate uncaging of 2, 5 and 10 spines at distal sites. In grey, expected compound EPSPs determined by arithmetically summing individual single spine responses. **C**, measured versus expected EPSP amplitudes for data shown in (B). Grey bar indicates range of expected EPSPs used to compute gain determined from mean ratio of measured vs expected EPSP amplitudes over 8-10mV range (grey box). Gain of distal input was similar in the absence (red dashed line) and presence (blue dashed line) of K^+ channel blockers (gain of 0.82±0.07 vs. 0.91±0.13 in the absence vs. presence of K^+ channel blockers, n=12; p=0.18, paired Wilcoxon signed rank test). Properties of individual single spine responses.

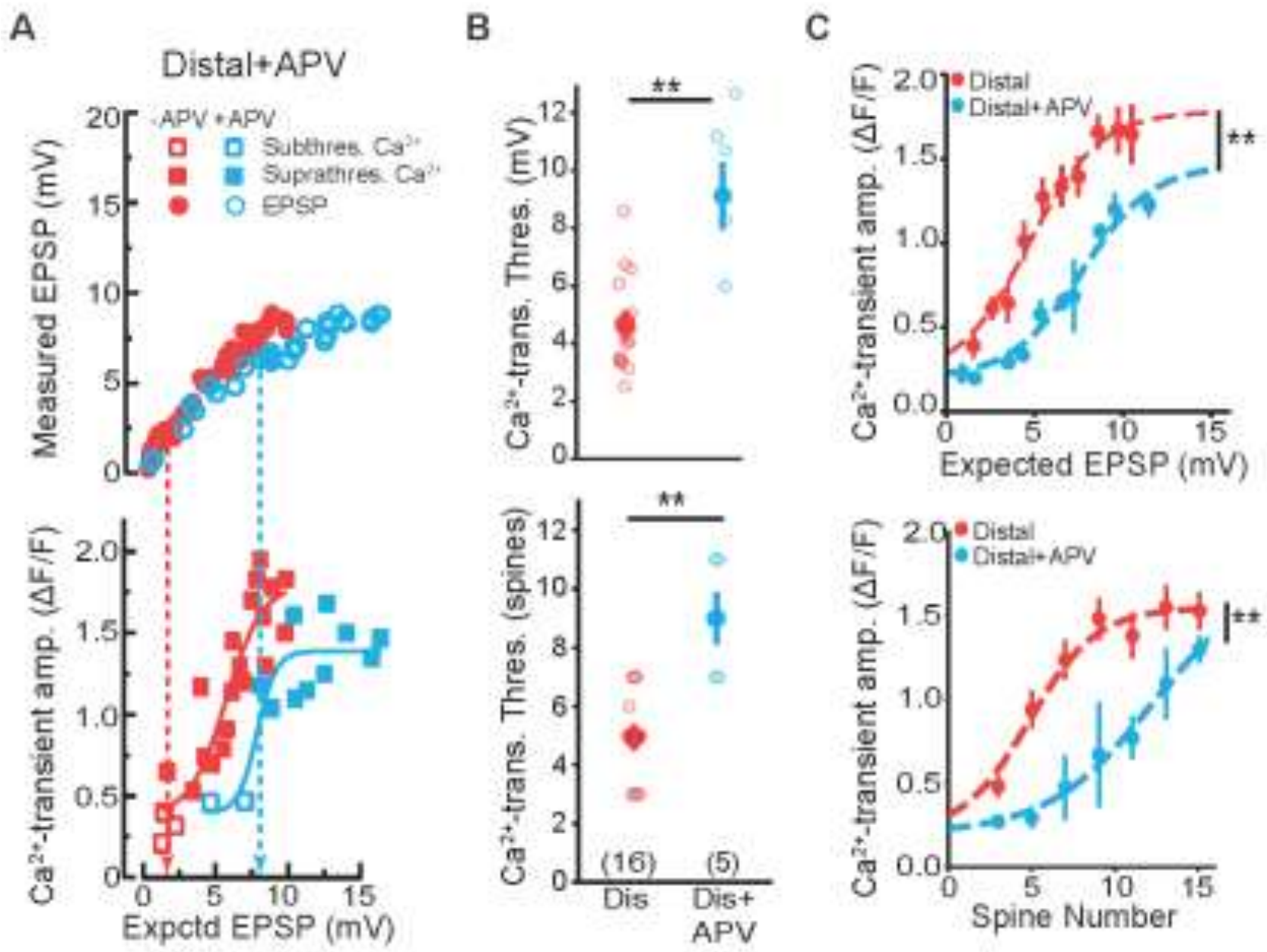
A
Distal+APV
-APV +APV
Subthres. Ca2+
Suprathres. Ca2+
EPSP
Measured EPSP (mV)
Ca2+-transient amp. (ΔF/F)
Expctd EPSP (mV)
B
Ca2+-trans. Thres. (mV)
Ca2+-trans. Thres. (spines)
(16)
(5)
Dis
Dis+ APV
C
Distal
Distal+APV
Expected EPSP (mV)
Spine Number

Supplementary Fig. S12: Distal NMDA receptors mediate calcium influx into distal dendrites. **A**, At distal sites, application of APV (blue) shifted threshold for suprathreshold Ca^{2+} responses to the right. **B,** Mean Ca^{2+} transient threshold measured as expected EPSP amplitude (mV) (from A) or spine number. Distal Ca^{2+} threshold significantly increased in the presence of APV. Statistical significance determined using one-way unpaired Student's t-test distal sites vs. distal sites +APV, $p<0.01$ for both panels. **C**, Summary plot showing Ca^{2+}-transient amplitude against expected EPSP amplitude or spine number. Mean Ca^{2+}-transient amplitudes with sem binned in 1 mV bins of expected EPSP amplitude or spine number. Ca^{2+}-transient amplitude were fit to sigmoid fits. Statistical significance determined using two-way ANOVA with Bonferroni's post-test. Main effect distal (n=9) vs. distal+APV (n=3): $F_{(1,4)} = 22.9$, $p<0.01$; spine number $F_{(1,4)}=11.2$, $p<0.01$; interaction: $F_{(1,4)}=0.8$, $p=0.51$; two-way ANOVA main effect distal (n=9) vs. distal+APV (n=3): $F_{(1,8)} = 34.7$, $p<0.01$; expected EPSP, $F_{(1,8)}=18.5$, $p<0.01$; interaction: $F_{(1,8)}=0.8$, $p=0.62$).

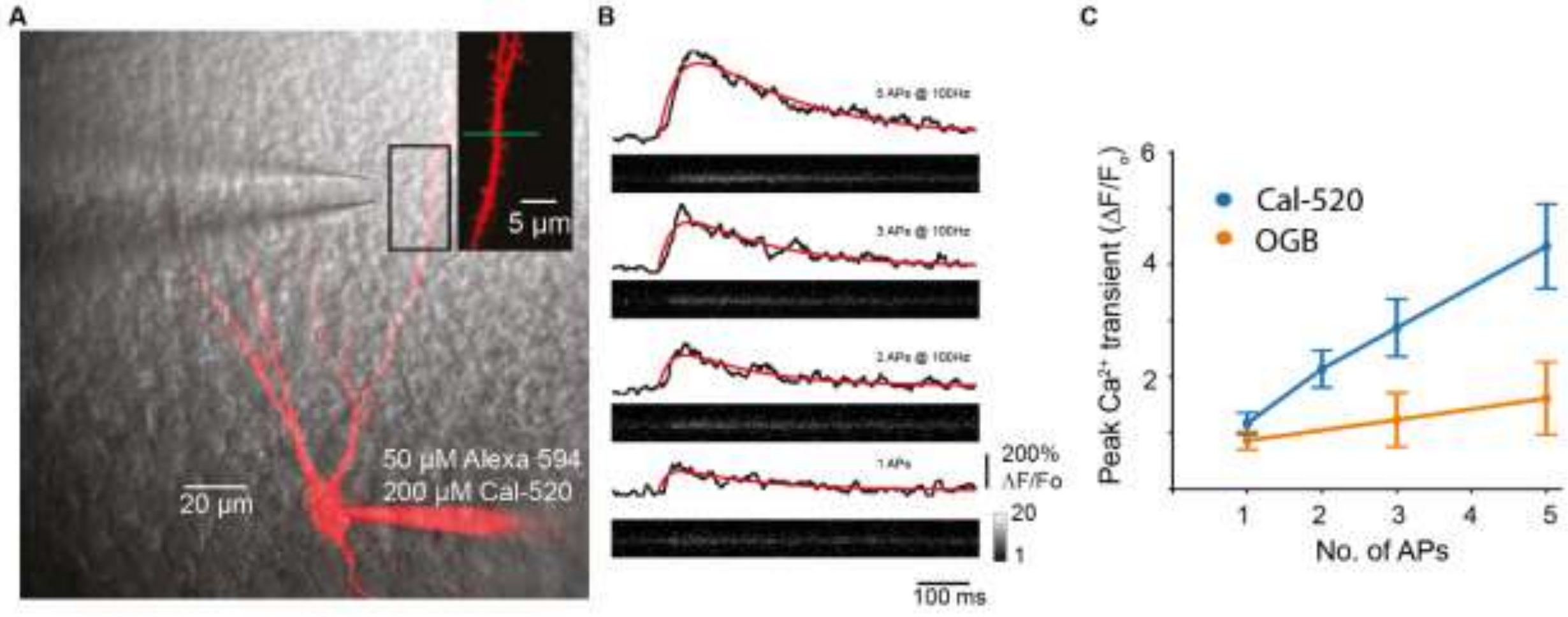

A
5 µm
50 µM Alexa 594
200 µM Cal-520
20 µm
B
5 APs @ 100Hz
3 APs @ 100Hz
2 APs @ 100Hz
1 APs
200%
ΔF/Fo
20
1
100 ms
C
Cal-520
OGB
Peak Ca2+ transient (ΔF/Fo)
6
4
2
1
2
3
4
5
No. of APs

Supplementary Fig. S13: Linear increases in Cal-520 fluorescence. **A**, Two-photon image of granule cell filled with Alexa 594 and calcium indicator Cal-520. Application pipette for MNI glutamate visible in transmitted channel. Intra-dendritic Ca^{2+} recorded at proximal dendrite. *Inset*: higher magnification image with line-scan position indicated (green line). **B**, Dendritic Ca^{2+} responses in response to somatically-evoked APs. **C**, Increasing the number of APs elicited linear increases in peak Ca^{2+} transient using both Cal-520 and OGB-1. Peak responses were significantly larger with Cal-520 and remained linear up to a DeltaF/F_o of at least 4.
